# FA-97, a New Synthetic Caffeic Acid Phenethyl Ester Derivative, Ameliorates DSS-Induced Colitis Against Oxidative Stress by Activating Nrf2/HO-1 Pathway

**DOI:** 10.3389/fimmu.2019.02969

**Published:** 2020-01-08

**Authors:** Yu Mei, Zihao Wang, Yifan Zhang, Ting Wan, Jincheng Xue, Wei He, Yi Luo, Yijun Xu, Xue Bai, Qi Wang, Yujie Huang

**Affiliations:** ^1^Science and Technology Innovation Center, Guangzhou University of Chinese Medicine, Guangzhou, China; ^2^Institute of Clinical Pharmacology, Guangzhou University of Chinese Medicine, Guangzhou, China; ^3^Centre of Clinical Research for Chinese Medicine, School of Chinese Medicine, Institute of Brain and Gut Axis (IBAG), Hong Kong Baptist University, Kowloon Tong, China; ^4^Department of Chemistry, Southern University of Science and Technology, Shenzhen, China; ^5^Southwestern Medical University Affiliated Chinese Medicine Hospital, Quzhou, China

**Keywords:** inflammatory bowel disease, oxidative stress, nuclear factor erythroid 2-related factor (Nrf2), reactive oxygen species (ROS), caffeic acid phenethyl ester (CAPE)

## Abstract

Inflammatory bowel disease (IBD) is a chronic idiopathic inflammatory disorder of gastro-intestinal tract, lacking effective drug targets and medications. Caffeic acid phenethyl ester (CAPE), a phenolic constituent derived from propolis, has been reported to be a potential therapeutic agent for IBD with low water solubility and poor bioavailability. In this study, we synthesized a new CAPE derivative (FA-97) and aimed to investigate the effect of FA-97 on DSS-induced colitis. Here, we found that FA-97 attenuated body weight loss, colon length shortening and colonic pathological damage in colitis mice, as well as inhibited inflammatory cell infiltration and expression of pro-inflammatory cytokines in colons. In addition, FA-97 reduced ROS production and MDA generation, while total antioxidant capacity both in DSS-induced colitis mice and LPS-stimulated primary BMDMs and RAW 264.7 cells were enhanced. Mechanically, FA-97 activated Nrf2 followed by increased HO-1 and NQO-1 and down-regulated nuclear levels of p65 and c-Jun, to suppress DSS-induced colonic oxidative stress. Moreover, FA-97 decreased pro-inflammatory cytokine expression and increased the antioxidant defenses in RAW 264.7 via Nrf2 activation. In general, this study reveals that FA-97 activates Nrf2/HO-1 pathway to eventually alleviate DSS-induced colitis against oxidative stress, which has potential activity and may serve as a candidate for IBD therapy.

## Introduction

Crohn's disease (CD) and ulcerative colitis (UC) are major forms of inflammatory bowel diseases (IBD), which are chronic idiopathic inflammatory disorders of the gastro-intestinal tract, causing a lifelong burden on patients (1). At the turn of the twenty first century, IBD has become a global disease with accelerating incidence in newly industrialized countries and the prevalence of IBD has also risen to more than 0.3% of the population in the developed countries (2). Clinically, IBD is characterized by severe abdominal pain, diarrhea, bleeding, fever and malnutrition reflecting the underlying inflammatory process (3). Up to now, the exact etiology of IBD is not yet fully clarified, caused by the interplay of various factors including genetic susceptibility, the commensal enteric flora, infectious agents, immune system dysfunction, and the external environment, in consequence, there is no definitive treatment (4). It is urgent to explore novel preventive intervention and therapeutics for IBD.

The inflammation conditions and uncontrolled immune system result in an increased oxidative burden due to the sustained overproduction of reactive oxygen species (ROS) by activated macrophages and neutrophils (5). Low level of ROS possess a protective effect via the activation of protective signaling pathways against inflammation, while high level of ROS creates oxidative stress leading to a loss of homeostasis with subsequent cellular oxidative stress damage, including lipid peroxidation products, protein modifications and the overproduction of pro-inflammatory cytokines (6). The elevated local level of pro-inflammatory cytokines, especially interleukin (IL)-1β, IL-6, IL-12, IL-17, IL-23, interferon-γ (IFN-γ), and tumor necrosis factor (TNF)-α, is an significant characteristic of the uncontrolled immune system in IBD (7), which can induced by ROS via the nuclear factor-κB (NF-κB) and nuclear transcription of activation protein-1 (AP-1) signaling (8). Therefore, scavenging ROS is considered to be crucial to relief the intestinal inflammation.

The nuclear factor erythroid 2-related factor 2 (Nrf2) is a redox-sensitive transcription factor playing an influential role in orchestrating cellular antioxidant response mechanisms (9). Kelch-like ECH-associated protein 1 (Keap1)-Nrf2-antioxidant response elements (ARE) pathway represents one of the most significant cellular defense mechanisms against oxidative stress (10). Under basal (reducing) conditions of cell growth, the cytoplasmic protein Keap1 constitutively targets Nrf2 for ubiquitin-dependent proteasomal degradation (11). However, following exposure to oxidative stress, Nrf2 escapes from Keap1-mediated degradation, translocates into nucleus and activates expression of a series of ARE-dependent genes encoding cytoprotective phase II detoxification and antioxidant enzymes, such as heme oxygenase-1 (HO-1), quinone oxidoreductase 1 (NQO1), glutathione peroxidase (GPx), and glutamate-cysteine ligase (GCL) (12). It was suggested that Nrf2 deficiency results in a higher susceptibility to IBD and colitis-associated colorectal cancer (13, 14). Moreover, the application of Nrf2 activator ameliorated DSS-induced acute and chronic colitis (15, 16). Thus, targeting Nrf2/HO-1 signaling has been considered as a sensible strategy in discovering preventive and therapeutic agents for IBD.

Caffeic acid phenethyl ester (CAPE) is a phenolic constituent and can be derived from honeybee propolis (17). It has been widely reported that CAPE possesses no known side effects (17) and could be a potential therapeutic agent for IBD by suppressing pro-inflammatory cytokines production and enhancing epithelial barrier function (18). However, the CAPE molecule can be decomposed easily in biological systems on account of its ester bond (α-β unsaturated carbonyl) and the catechol groups (19). It has been reported that the application of CAPE *in vivo* is also under restrictions due to the low water solubility (20). Moreover, the poor bioavailability of CAPE limit its efficacy and the half-life of CAPE is 20–28 min independent of the dose after intragastric administration (21).

**Figure 1:**
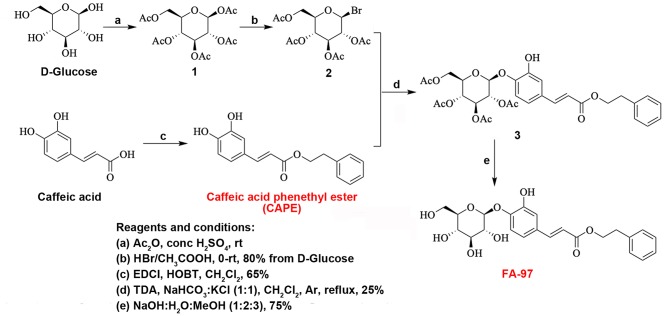
Synthesis scheme of FA-97. FA-97 (caffeic acid phenethyl ester 4-*O*-glucoside, C_23_H_26_O_9_, MW = 446.16 g/mol) was synthesized via the coupling reaction between a acetyl protected brominated D-glucose and caffeic acid phenethyl ester (CAPE) starting from commercially available caffeic acid. Reagents and conditions of each chemical step are shown in the left bottom of the figure.

In this study, caffeic acid phenethyl ester 4-*O*-glucoside (FA-97) (Figure 1), a new CAPE derivative, was synthesized via the coupling reaction between a acetyl protected brominated D-glucose and CAPE starting from commercially available caffeic acid (Figure 1). With the deprotection of coupled key intermediate, FA-97 was synthesized in good yields and owns better water solubility than CAPE. In addition, FA-97 was found to ameliorated DSS-induced colitis against oxidative stress and inhibited pro-inflammatory cytokines production both *in vivo* and *in vitro*. Further mechanism researches revealed that FA-97 exerted anti-inflammation ability by suppressing NF-κB and AP-1 pathways via the activation of Nrf2/HO-1 signaling.

## Materials and Methods

### Compounds and Reagents

FA-97 (caffeic acid phenethyl ester 4-*O*-glucoside, C_23_H_26_O_9_, MW = 446.16 g/mol) (>99% purity) was synthesized via the coupling reaction between a acetyl protected brominated D-glucose and caffeic acid phenethyl ester (CAPE) starting from commercially available caffeic acid. D-glucose was dissolved in anhydrous Ac_2_O and conc. H_2_SO_4_ at 0°C. Then the solution was allowed to room temperature and stirred overnight. Water and EtOAc were added at 0°C and the resulted mixture was then extracted with EtOAc. The combined organic layers were dried over Na_2_SO_4_, concentrated under reduced pressure (Figure 1). FA-97 was dissolved in dimethylsulfoxide (DMSO) as stock solution at 0.1 M and stored at −20°C. FA-97 stock solution was diluted with culture medium freshly to the final concentration before each experiment *in vitro* and the final DMSO concentration did not exceed 0.1% with no effect on cell viability. In the *in vivo* study, FA-97 was prepared daily with 3% Tween-80 as intragastric administration.

5-Aminosalicylic acid (5-ASA), caffeic acid phenethyl ester (CAPE), LPS (*E. coli*: Serotype O55:B5) and tin protoporphyrin-IX (SnPP, an HO-1 inhibitor) were purchased from Sigma-Aldrich (St. Louis, MO, USA). Dextran sulfate sodium (DSS, MW = 36–50 kDa) was obtained from MP Biomedicals Inc. (Irvine, CA, USA). Dye DAPI was purchased from Invitrogen (Carlsbad, CA, USA). Bovine serum albumin (BSA) was purchased from Roche Diagnosis (Shanghai) Ltd. (Shanghai, China).

### Cell Culture

The RAW 264.7 cell line was purchased from the Cell Bank of Shanghai Institute of Biochemistry & Cell Biology at the Chinese Academy of Sciences (Shanghai, China) and cultured in DMEM supplemented with 10% (v/v) fetal bovine serum (FBS), 100 U/ml penicillin and 100 U/ml streptomycin, in a stable environment with 5% CO_2_ at 37°C. Before used in the following *in vitro* experiments, RAW 264.7 cells were treated with FA-97 (0, 0.25, 0.5, and 1 μM) for 24 h, followed by stimulation with LPS (1 μg/ml) for another 2 h.

### Isolation of Bone Marrow Derived Macrophages (BMDMs)

The BMDMs were isolated from C57BL/6 mice and cultured with DMEM supplemented with 10% FBS, granulocyte-macrophage colony-stimulating factor (GM-CSF, 20 ng/mL), 100 U/ml penicillin and 100 U/ml streptomycin. The bone marrow cells were harvested and seeded on cell culture dishes (60 mm × 15 mm). After exchanging the culture fluid every 3 days within about 1 week, the adherent macrophages were obtained. By being cultured for another 6 h without GM-CSF, the cells were used as BMDMs in the following experiments.

### DSS-Induced Colitis and Experimental Design

The female C57BL/6 mice (35–40 days, 18–22 g) were supplied by Shanghai Laboratory Animal Center, China Academy of Sciences (Shanghai, China). Experimental protocols were in accordance with National Institutes of Health regulations and approved by the Institutional Animal Care and Use Committee. Throughout the acclimatization and study periods, all animals had access to food and water *ad libitum* and were maintained on a 12 h light/dark cycle (21 ± 2°C with a relative humidity of 45 ± 10%). The mice were randomly assigned to control, DSS-treated, DSS + FA-97-treated (2.5, 5 and 10 mg/kg), DSS + 5-ASA-treated (80 mg/kg) and DSS + CAPE-treated (30 mg/kg) groups. Acute colitis in mice was induced by adding 5% (w/v) DSS to their drinking water and allowing them drink *ad libitum* for 7 days (from day 5 to day 11) and thereafter provided with regular water for another 3 days (from day 12 to day 14). Mice in control group were received drinking regular water in the whole experiment. To assess the chemoprevention effect of FA-97 on DSS-induced colitis in mice, FA-97, CAPE and 5-ASA were given intragastrically from day 1 to day 14 (terminal of the experiment), respectively. For mechanism study, mice were intraperitoneally injected with ML385 (purchased from Selleck Chemicals) 30 mg/kg/day (dissolved in Cremophor EL) for 5 days.

### Assessment of Colonic Inflammation

To evaluate the clinical symptoms of DSS-induced colitis, all mice were weighed and inspected for diarrhea and rectal bleeding daily. The disease activity index (DAI) was calculated according to a standard scoring system as described previously (22), which is the combined score of body weight loss, stool consistency and rectal bleeding. Briefly, the DAI scores are defined as follows: loss in body weight (0 = no loss, 1 = 5–10%, 2 = 10–15%, 3 = 15–20%, 4=>20%); rectal bleeding (0 = no blood, 2 = positive, 4 = gross blood); appearance of diarrhea (0 = none, 2 = mild, 4 = gross diarrhea).

After colitis induction, animals were sacrificed on day 15. The entire colon of each mouse was removed for length measurement and the colon samples were opened longitudinally, washed with normal saline. Segments (0.5 cm) from the distal colon were obtained, placed in a 1.5 ml cryogenic tube immediately and frozen in liquid nitrogen, and then stored at −80°C until further used in the following experiments (e.g., enzymatic activity measurements, ELISA, RT-PCR, and WB).

### Histological Analysis

To quantify the extent of mucosal damage, the histological analysis was performed. A 0.5 cm segment from the distal colon of mice was immersed in 4% paraformaldehyde (pH 7.4) for 24 h, embedded in paraffin, cut into 4 μm sections using standard histological techniques to prepare paraffin sections (for further analysis, e.g., Immunohistochemistry and immunofluorescence assay). The prepared paraffin sections were stained with hematoxylin and eosin (H&E), observed and photographed with a bright-field microscope (Leica Microsystems, Heerbrugg, Swiss).

### Single-Cell Preparation and FACS Analysis

The distal colon tissues (0.5 cm) were taken, cut into 5 mm pieces, transferred into 50 ml conical tubes and incubated in 20 ml in Hanks' balanced salt solution (10% FBS, 100 U/ml penicillin, 100 μg/ml streptomycin and 2 mM EDTA) at 37°C, 250 rpm/min for 1 h. Cell suspensions were passed through a 70 μm filter and the remaining colon tissues were washed with PBS, cut into 1 mm pieces and incubated with Hanks' balanced salt solution (5% FBS, 100 U/ml penicillin, 100 μg/ml streptomycin and 1 mg/ml type VIII collagenase) at 37°C, 250 rpm/min for 30 min, and vortexed for 20 s at the start, middle and end of incubation. Cell suspensions were passed through a 70 μm filter, pelleted by centrifugation at 1,500 rpm/min for 10 min at 4°C, washed with PBS, and then diluted down to 5–10 × 10^6^ cells/ml. For FACS analysis, single-cell suspensions were washed in FACS buffer (PBS, 0.1% BSA) and stained by the following panel of monoclonal antibodies to the cell surface molecules: allophycocyanin (APC)-conjugated anti-CD11b (clone M1/70, catalog # ab25482, Abcam) purchased from Abcam, Inc. (Cambridge, UK), fluorescein isothiocyanate (FITC)-conjugated anti-F4/80 (clone BM8, eBioscience) and phycoerythrin (PE)-conjugated anti-Gr-1 (clone RB6-8C5, eBioscience) obtained from eBioscience (San Diego, CA, USA). Cells were washed in FACS buffer and detected using an LSRII flow cytometer (Becton Dickinson, Franklin Lakes, NJ). Data were analyzed using flowjo7 software (Tree Star, Ashland, OR). CD11b^+^F4/80^+^ cells appeared to be monocytes/macrophages, and CD11b^+^Gr-1^+^ cells appeared to be monocytes/neutrophils, which accords with the previous study (23).

### Immunofluorescence (IF) of Colon Tissues

Immunofluorescence analysis was performed to test the CD11b positive inflammatory cell infiltration in colon tissues. Briefly, paraffin-embedded colon tissue sections were prepared as described above and followed by being deparaffinized, rehydrated and washed in PBS. After being treated with 3% hydrogen peroxide and blocked with 5% BSA, the colon tissue sections were incubated at room temperature with FITC-conjugated anti-CD11b (1:100, clone M1/70, catalog # ab8878, Abcam) for 1 h. Then the slides were then counter-stained with DAPI (1:1000). at room temperature in dark for 30 min. The reaction was stopped by washing in water gently for 3 min. The confocal laser-scanning microscope (Olympus, Tokyo, Japan) was used to acquire images. Settings for image acquisition were identical for control and experimental tissues.

### Myeloperoxidase (MPO) and Inducible Nitric Oxide Synthase (iNOS) Activity Measurement

For MPO and iNOS activity measurement, 0.5 cm colonic samples stored at −80°C were taken from the distal region and cut into pieces. Colon pieces were homogenized in ice-cold potassium phosphate buffer (100 mmol/l, pH = 7.4), sodium orthovanadate (10 mM), PMSF (100 mM) and protease inhibitor cocktail (Sigma-Aldrich, St. Louis, MO, USA) with a FastPrep™24 homogenizer, and centrifuged at 12,000 rpm/min for 30 min at 4°C. The supernatant was taken to assess the MPO and the iNOS activities according to manufacturer's instructions. O-dianisidine method was used in the MPO activity assessment to quantify the neutrophil infiltration into inflamed colonic mucosa. The MPO activity assay kit and the Nitric Oxide Synthase Assay Kit were purchased from Nanjing Jiancheng Bioengineering Institute (Nanjing, China). Protein concentration of each sample was determined by bicinchoninic acid (BCA) protein assay kit (Thermo, MA, USA). MPO and iNOS activities were measured by a standard curve of samples in units of MPO/mg or iNOS/mg of protein.

### Immunohistochemistry

Distal colonic segments (0.5 cm) from mice in each group were taken and the colonic paraffin sections were prepared as described above (Histological analysis). The immunohistochemistry assay used for testing the expression of IL-1β, IL-6, TNF-α, p65, c-Jun, HO-1, and Nrf2 of the colonic tissues was performed according to the manufacture instruction in the Immunohistochemistry Application Solutions Kit (#13079, Cell Signaling Technology, Danvers, MA, USA). Briefly, colonic paraffin sections were deparaffinized by being incubated in sequence in three washes of xylene for 5 min each, two washes of 100% ethanol for 10 min each, two washes of 95% ethanol for 10 min each, and then washed twice in dH_2_O for 5 min each. Bring slides to a boil in 10 mM sodium citrate buffer (pH = 6.0) and maintain at a sub-boiling temperature for 10 min. Cool slides on bench top for 30 min and wash three times for 5 min each. After being incubated in 3% hydrogen peroxide for 10 min, sections were blocked with 100 μl blocking solution for 1 h at room temperature and stained with the following primary antibodies: rabbit monoclonal anti-IL-1β and anti-p65 antibodies (1:100, Cell Signaling Technology, Danvers, MA, USA); rabbit monoclonal anti-IL-6, anti-TNF-α, anti-c-Jun, anti-HO-1, and anti-Nrf2 antibodies (1:200, Abcam, Cambridge, United Kingdom). After incubation in a humidified chamber overnight at 4°C, the tissue sections were washed with PBS three times, covered with 2 drops SignalStain® Boost Detection Reagent for 30 min at room temperature, and washed three times with PBS for 5 min each. Apply 200 μl SignalStain® DAB to each section for 8 min, immerse slides in dH_2_O and washed twice, and then dehydrate sections by incubating in 95% ethanol for 10 s twice, repeating in 100% ethanol for 10 s twice and in xylene for 10 s twice. Colon sections were observed and photographed with a bright-field microscope (Leica Microsystems, Heerbrugg, Swiss).

### Enzyme-Linked Immunosorbent Assay (ELISA)

Colon tissue homogenates were obtained as described above (MPO activity measurement). The amount of total extracted protein was determined by a BCA protein assay kit (Thermo, MA, USA). RAW 264.7 cells or BMDMs were treated with FA-97 (0, 0.25, 0.5, and 1 μM) for 24 h, followed by stimulation with LPS (1 μg/ml) for another 2 h and the culture supernatant was collected. The concentrations of IL-1β, IL-6, TNF-α, and IL-12 in the colon homogenate or in the culture supernatant of RAW 264.7 cells and BMDMs were determined according to the manufacture instructions in Duo-set enzyme linked immune sorbent assay kits, purchased from R&D Systems Co. Ltd. (Minneapolis, MN, USA).

### Reactive Oxygen Species (ROS) Assay

The assay was performed to analyze the levels of ROS in the colon cells and RAW 264.7 cells by using fluorescent dye 2′7′-dichlorofluorescein-diacetate (DCFH-DA, S0033, Beyotime Institute of Biotechnology, Shanghai, China). The non-fluorescent DCFH-DA can be oxidized to fluorescent 2′7′-dichlorofluorescein (DCF) by ROS. Twenty milligram colonic samples from the distal colon of mice in each group were taken, cut into pieces and incubated with Hanks' balanced salt solution with 1 mg/ml type VIII collagenase at 37°C, 250 rpm/min for 30 min, and vortexed for 20 s at the start, middle and end of incubation. Cell suspensions were passed through a 70 μm filter and colon cells were collected by centrifugation at 1,500 rpm/min for 10 min at 4°C. The collected colon cells and RAW 264.7 cells were incubated with DCFH-DA for 30 min at 37°C in the dark. The conversion of DCFH-DA to DCF was assessed by a spectrofluorimeter at an excitation wavelength of 488 nm and an emission wavelength of 525 nm. Parallel blanks were used to standardize DCF. ROS level were quantified by a DCF standard curve. RAW 264.7 cells on coverslips were fixed with 4% formaldehyde, incubated with DCFH-DA for 20 min at 37°C in the dark, washed with medium three times to remove the extra DCFH-DA, and then photographed by fluorescence microscope (Leica Microsystems, Heerbrugg, Swiss).

### Measurement of Malondialdehyde (MDA) Level and Total Antioxidant Capacity

0.5 cm distal colon tissues of mice in each group were taken, cut into pieces and homogenized in ice-cold potassium phosphate buffer as described previously. RAW 264.7 cells were collected and homogenized in cold PBS. The MDA level and total antioxidant capacity were measured according to the manufacturer's instructions of the MDA assay kit (S0131, Beyotime Institute of Biotechnology, Shanghai, China) and the total antioxidant capacity assay kit (Beyotime Institute of Biotechnology, S0121, Shanghai, China), respectively. The total protein content was determined by BCA protein kit (Thermo, MA, USA).

### Real-Time PCR Analysis

Total RNA was extracted using Total RNA Extraction Reagent (Vazyme, China). cDNA was made using 500 ng of total RNA with Hiscript® II Reverse Transcriptase (Vazyme, China). qPCR incubations were run with 200 nM of gene-specific primers and HiScript® II Q RT SuperMix for qPCR (Vazyme, China). The relative amount of target mRNA was determined using the comparative threshold (Ct) method by normalizing target mRNACt values to those for β-Actin (ΔCt). The qPCR primer sequences were described as follows:

Mouse IL-1β-sense (5′-CCAAGCTTCCTTGTGCAAGTA-3′);

Mouse IL-1β-antisense (5′-AAGCCCAAAGTCCATCAGTGG-3′);

Mouse IL-6-sense (5′-ACAACCACGGCCTCCCTAC-3′);

Mouse IL-6-antisense (5′-TCTCATTTCCACGATTTCCCAG-3′);

Mouse TNF-α-sense (5′-ATGAGCACAGAAAGCATGATCCGC-3′);

Mouse TNF-α-antisense (5′-AAAGTAGACCTGCCCGGTC-3′);

Mouse IL-12-sense (5′-GGAAGCACGGCAGCAGAATA-3′);

Mouse IL-12-antisense (5′-AACTTGAGGGAGAAGTAGGAATGG-3′);

Mouse MIP-1α-sense (5′-GCTGACTACTTTGAGACGAGC-3′);

Mouse MIP-1α-antisense (5′-CCAGTCCATAGAAGAGGTAGC-3′);

Mouse IL-17-sense (5′-CTGGACTCTCCACCGCAATG-3′);

Mouse IL-17-antisense (5′-CACCAGCATCTTCTCGACCC-3′);

Mouse β-Actin-sense (5′-TGCTGTCCCTGTATGCCTCT-3′);

Mouse β-Actin-antisense (5′-TTTGATGTCACGCACGCACGATTT-3′).

### Preparation of Cytosolic and Nuclear Extracts

Nuclear and cytosolic protein extracts were prepared according to the user guide of Nuclear and Cytoplasmic Protein Extraction Kit (Beyotime Institute of Biotechnology, Shanghai, China). After separated, cytosolic and nuclear fractions were subjected to Western Blot analysis. Final detection was performed with Western Blot analysis according to the modified method as described below.

### Western Blot Analysis

The colonic tissue was minced and homogenized in ice-cold potassium phosphate buffer described above. For collecting the cell protein sample, cells were harvested and lysed by RIPA buffer (50 mM TrisCl (pH 7.6), 150 mM NaCl, 1 mM EDTA, 1% (m/v) NP-40, 0.2 mM PMSF, 0.1 mM NaF and 1.0 mM DTT) incubation on ice for 40 min. The colonic tissue homogenate and the cell lysis were then clarified by centrifuging at 12,000 rpm/min for 20 min at 4°C, and the concentration of protein in the supernatants was detected using BCA assay. Equal amounts of protein (60 μg) were loaded onto a 5 and 10% gel, subjected to SDS-PAGE, and electro-transferred onto polyvinylidene difluoride (PVDF) membranes (Hybond-P PVDF Membrane, Amersham Biosciences, Buckinghamshire, UK). The membranes were blocked with 3% BSA in PBS with 0.1% Tween 20 (PBST) at room temperature for 1.5 h and incubated with the indicated primary antibodies overnight at 4°C. Primary antibodies against HO-1, NF-κB (p65), p-p65 (Ser 536), IκBα, p-IκBα (Ser 32), c-Fos, p-c-Fos (Ser 32), and Lamin A were obtained from Cell Signaling Technology (Danvers, MA, USA) (1:500 dilution); antibodies against c-Jun, p-c-Jun (Ser 63), Nrf2, and NQO-1 were purchased from Abcam, Inc. (Cambridge, UK) (1:800 dilution); antibody against β-actin and GAPDH were purchased from Santa Cruz Biotechnology (Santa Cruz, CA, USA) (1:1000 dilution). Membranes were washed with PBST for three times and incubated with horseradish peroxidase (HRP)-conjugated anti-mouse or anti-rabbit IgG (1:2000, Cell Signaling Technology) at room temperature for 1.5 h. The immune complexes were detected by the ECL chemiluminescence method (Termo Fisher Scientific). All blots were stripped and re-probed with β-actin, GAPDH or Lamin A antibody to ascertain equal loading of proteins.

### Immunofluorescence Staining

RAW 264.7 cells were grown on coverslips and treated with FA-97 (1 μM) for 24 h, followed by stimulation with LPS (1 μg/ml) for another 2 h. Cells on coverslips were fixed with 4% paraformaldehyde, permeabilized in 0.2% Triton X-100 and incubated with 3% BSA. After incubated with rabbit monoclonal anti-NF-κB (p65) antibody (1:100, Cell Signaling Technology, Danvers, MA, USA) or anti-c-Jun antibody (1:100, Abcam, Inc., Cambridge, UK) in a humidified chamber overnight at 4°C, cells were exposed to Alexa Fluor® 488 conjugate anti-rabbit IgG (1:2000, Cell Signaling Technology, Danvers, MA, USA) for 30 min at room temperature and stained with DAPI (1:1000, Invitrogen, Carlsbad, CA, USA). Cells were observed and photographed with a confocal laser scanning microscope (Fluoview FV 1000, Olympus, Tokyo, Japan).

### Luciferase Reporter Assay

The transcriptional activity of Nrf2 was determined using ARE Reporter kit (BPS Bioscience, San Diego, CA, USA). Briefly, RAW 264.7 cells cultured in a 24-well plates were co-transfected for 24 h with ARE luciferase reporter plasmid (3 μg) and a plasmid that constitutively expressed Renilla luciferase (0.3 μg) using Lipofectamine™ 2000 (Invitrogen; Thermo Fisher Scientific, Inc.). After serum recovery, cells were treated with FA-97 (0.25, 0.5, and 1 μM) for 24 h, followed by LPS (1 μg/ml) stimulation for 2 h. The cells were then lysed with 1 × Passive Lysis Buffer (100 μl/well) for 20 min at room temperature. The lysates were centrifuged, and the supernatants were harvested. Thereafter, the ARE-luciferase activities were determined using a luciferase assay kit in accordance with the manufacturer's instructions (Promega, Madison, WI, USA). Data were normalized with Renilla luminescence. The data were obtained from three independent experiments and expressed as the inducible fold change compared to the vehicle control (cells treated with 0.1% DMSO).

### Transfection of Nrf2 siRNA

Nrf2 siRNA sequence was purchased from (Thermo Fisher Scientific, Hudson, NH, United States). RAW264.7 cells were plated in six-well plates with fresh medium to be 60% confluent at transfection. Nrf2 siRNA or non-targeting siRNA (NT siRNA) transfection were performed according to the manufacturer's instructions of Lipofectamine 2000 reagent (Invitrogen, Carlsbad, CA, USA). Briefly, dilute Lipofectamine® Reagent (3 μl) in Opti-MEM ® medium (150 μl), dilute 3 μl siRNA (3 μg/μl) in Opti-MEM® medium (150 μl), and add diluted siRNA to diluted Lipofectamine® 2000 Reagent (1:1 ratio). After incubated at room temperature for 5 min, the siRNA-lipid complex (300 μl per well) was added to RAW 264.7 cells. After incubated for another 24 h at 37°C, cells were treated with FA-97 (1 μM) for 24 h and followed by LPS (1 μg/ml) stimulation for 2 h. Then the transfected cells were used for further analyzed.

### Statistical Analysis

Data shown in the study were obtained from at least three independent experiments and all data in different experimental groups were expressed as the mean ± SD. Statistical analyses were performed using a One-Way ANOVA, with *post-hoc* analysis. Details of each statistical analysis are provided in figure legends. Differences with *P* < 0.05 were considered statistically significant.

## Results

### FA-97 Attenuates DSS-Induced Colitis in Mice

**Figure 2:**
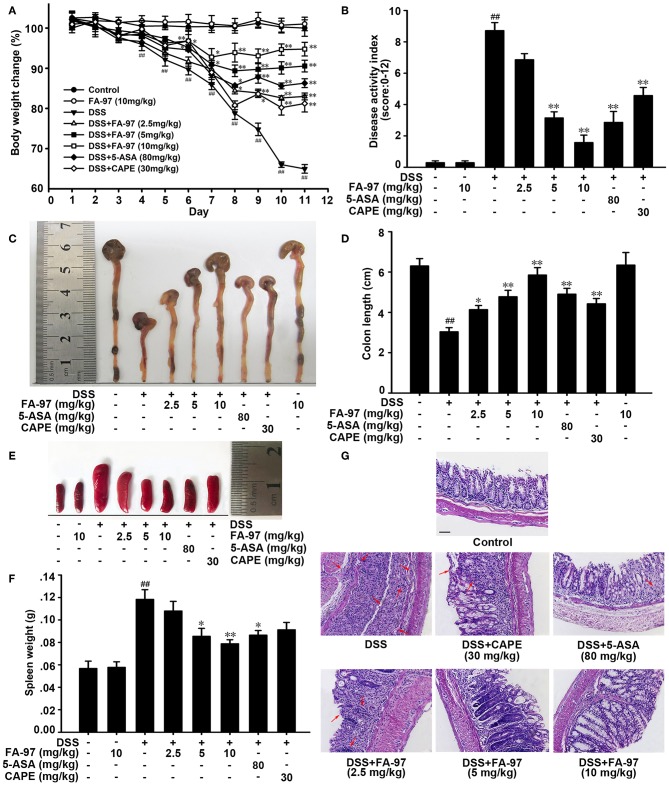
Effect of FA-97 on DSS-induced experimental colitis. **(A)** Body weights of mice in each group (*n* = 8) were measured. The body weight was expressed as a percentage of weight change for each individual mouse and was calculated relative to the starting body weight on day 1. **(B)** Disease activity index (DAI) of mice in each group. **(C)** Macroscopic appearance of the representative colon from each group. **(D)** The quantification of colon length from each group of mice. **(E)** Macroscopic appearance of the representative spleen from each group. **(F)** The quantification of spleen weight of mice from each group. **(G)** Representative images showing colon pathologic abnormalities with hematoxylin and eosin (H&E) staining. Scale bars, 100 μm. Data are presented as mean ± SD. ^##^*P* < 0.01 compared with control group and *P < 0.05, **P < 0.01 compared with DSS-treated group.

The colitis mice induced by DSS is a well-established preclinical model exhibiting a majority of phenotypic features associated with human inflammatory bowel disease (24), which was adopted here to assess the effect of FA-97 on colitis *in vivo*. The dramatic body weight loss, diarrhea, rectal bleeding, and colon shortening are typical symptoms of DSS-induced severe inflammation. As shown in Figure 2A, FA-97 attenuated the profound weight loss of DSS-induced mice in a dose dependent manner. The scores of disease activity index (DAI), a combined score with the incidence of weight loss, diarrhea and rectal bleeding, was then calculated according to a standard scoring system (22). Compared with control group, the DAI of DSS-induced mice is 8.7, FA-97 (10 mg/kg)-treated mice showed recovery from colitis with a DAI of 1.5 (Figure 2B). Moreover, the colon shortening was improved by FA-97 (Figures 2C,D), and FA-97 antagonized DSS-induced gain of spleen weight dose-dependently (Figures 2E,F). However, CAPE (30 mg/kg) showed no effect on the gain of spleen weight induced by DSS. In addition, the Haematoxylin & Eosin (H&E) staining with histopathological analysis was performed to determine the effect of LIG on DSS-induced colon injury. As shown in Figure 2G, compared with control group, DSS-induced colitis group exhibited significant erosion of the intestinal mucosa and lamina propria accompanying by the glandular epithelium disappearance and infiltration of a great many inflammatory cells, while FA-97 (10 mg/kg) inhibited inflammatory cell infiltration and preserved intact colonic architecture without apparent ulcer. These results indicate that FA-97 treatment restore colon damage and symptomatic features of DSS-induced colitis mice.

### FA-97 Diminishes DSS-Induced Inflammatory Cell Infiltration in Colon

**Figure 3:**
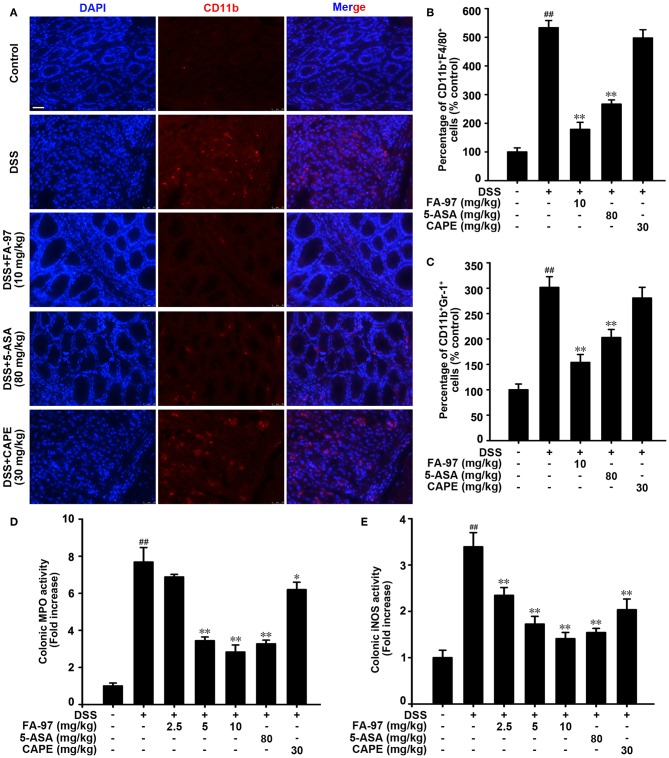
Effect of FA-97 on DSS-induced inflammation in colon. **(A)** Sections of colonic tissue were immunostained with DAPI (blue) and anti-CD11b-FITC (green) antibodies, and then observed by confocal laser-scanning microscope. **(B)** The distribution of CD11b^+^F4/80^+^ monocyte/macrophages and **(C)** CD11b^+^Gr-1^+^ monocytes/neutrophils in colonic tissues was detected by representative FACS blots. **(D)** The MPO activity in colonic tissues was detected by MPO Activity Assessment Kit using the O-dianisidine method. **(E)** The iNOS activity was measured by Nitric Oxide Synthase Assay Kit. Each experiment was performed at least three times and data are presented as mean ± SD. Scale bars, 25 μm. ^##^*P* < 0.01 compared with control group and *P < 0.05, **P < 0.01 compared with DSS-treated group.

Immune response in IBD is dependent on trafficking inflammatory cells to the gastrointestinal tract, which is regarded as inflammatory cell infiltration (25). As mainly expressed on the surface of leukocytes such as monocytes, neutrophils and macrophages, CD11b was used usually as a marker for monitoring the process of inflammatory cell infiltration (26). To evaluate the effect of FA-97 on DSS-induced inflammatory cell infiltration in colon, immunofluorescence staining with CD11b antibody was performed. As shown in Figure 3A, there was a mass of CD11b positive inflammatory cells accumulated at lesion site of the intestinal mucosa in DSS-treated mice, while FA-97 reduced the number of infiltrating CD11b^+^ inflammatory cells significantly. Additionally, FACS analysis showed that compared to DSS-treated group, FA-97 treatment decreased the number of CD11b and F4/80 positive macrophages as well as CD11b and Gr-1 positive monocytes/neutrophils in colon tissues (Figures 3B,C). However, compared with DSS-treated group, CAPE (30 mg/kg) had no effect on the CD11b^+^ expression in colonic tissues (Figures 3A–C). Myeloperoxidase (MPO) activity is a marker of neutrophil infiltration and the inducible nitric oxide synthase (iNOS) regulates NO release to damage intestinal mucosal cells and submucosa, which are referred as two typical indexes of inflammation damage (27). As a result, DSS-induced hyper-activated MPO (Figure 3D) and iNOS activity (Figure 3E) in colonic tissues were both inhibited by FA-97. Taken together, FA-97 diminished colonic inflammatory cells infiltration in DSS-induced colitis mice.

### FA-97 Inhibits Pro-inflammatory Cytokine Production and Enhances the Antioxidant Defenses in DSS-Induced Colitis Mice

**Figure 4:**
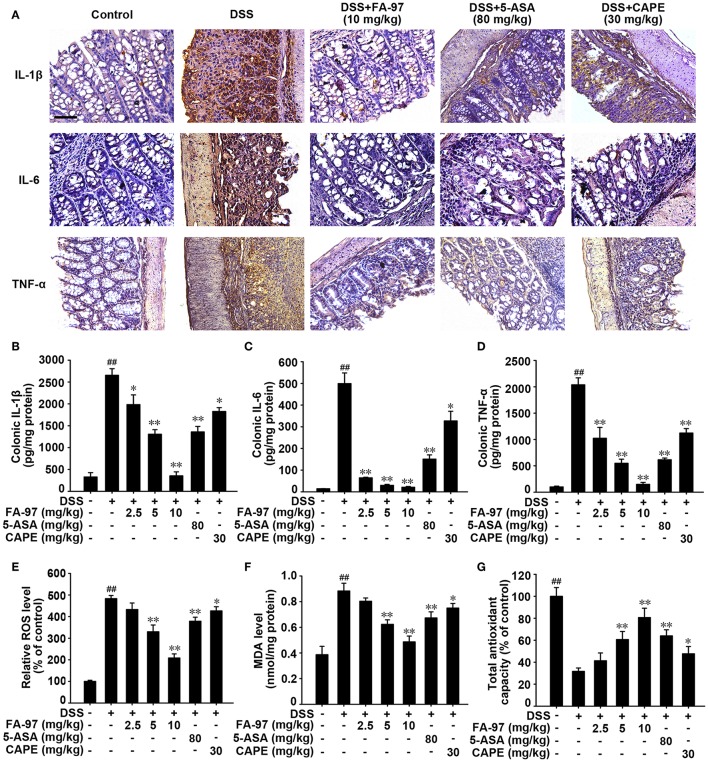
Effect of FA-97 on pro-inflammatory cytokine production and oxidative stress in DSS-induced colitis mice. **(A)** The expression of IL-1β, IL-6, and TNF-α in colon sections were detected by immunohistochemistry (IHC) and the positive cells were brown. **(B–D)** The level of IL-1β, IL-6, and TNF-α in colonic homogenate were measured by ELISA. **(E)** The colon cells were collected and incubated with DCFH-DA for 30 min at 37°C in the dark, and then the level of ROS was measured by spectrofluorimeter. **(F,G)** Colonic tissues were homogenized in cold PBS. The MDA level **(F)** and the total antioxidant capacity **(G)** in colon were measured according to the kit manufacturer's instructions. Each experiment was performed at least three times. Scale bars, 200 μm. Data are presented as mean ± SD. ^##^*P* < 0.01 compared with control group and *P < 0.05, **P < 0.01 compared with DSS-treated group.

The increased pro-inflammatory cytokines production in colon has been reported playing a influential role in the pathogenetic process of DSS-induced colitis (28). We assessed the level of several main pro-inflammatory cytokines in colons from DSS-induced colitis treated with FA-97. Immunohistochemistry analysis demonstrated that the increased number of IL-1β, IL-6, and TNF-α-positive cells (brown stained) in DSS-induced colonic mucosa was decreased by FA-97 (10 mg/kg) obviously (Figure 4A). ELISA assay showed that the level of IL-1β, IL-6, and TNF-α in colonic homogenates was reduced by FA-97 (Figures 4B,D). Moreover, RT-PCR showed that DSS-induced mRNA level of IL-1β, IL-6, TNF-α, IL-12, MIP-1α, and IL-17 in colonic tissue were down-regulated by FA-97 (Figure S1). Besides, compared with 5-ASA or CAPE, FA-97 was more effective in suppressing the expression and mRNA level of the pro-inflammatory cytokines in colons (Figures 4A–D and Figure S1).

During the development of IBD, oxidative stress and the process of colonic inflammation and are tightly linked, in which ROS overproduction could cause oxidative damage to cells and colon tissue (29). Thus, we evaluated the effect of FA-97 on oxidative stress in DSS-induced colitis mice. As shown in Figure 4E, compared with DSS-induced group, ROS generation in colon tissues from mice in FA-97-treated group was reduced. In addition, the elevated malondialdehyde (MDA), a gross indicator of lipid peroxidation induced by ROS, was counteracted by FA-97 treatment markedly (Figure 4F). Moreover, the dramatically reduced total antioxidant capacity in DSS-treated mice was restored by FA-97 in a dose-dependent manner (Figure 4G). In summary, FA-97 inhibited the production of pro-inflammatory cytokines and mitigated the oxidative stress in DSS-induced colitis mice.

### FA-97 Activates Nrf2/HO-1 Signaling in DSS-Induced Colitis Mice

**Figure 5:**
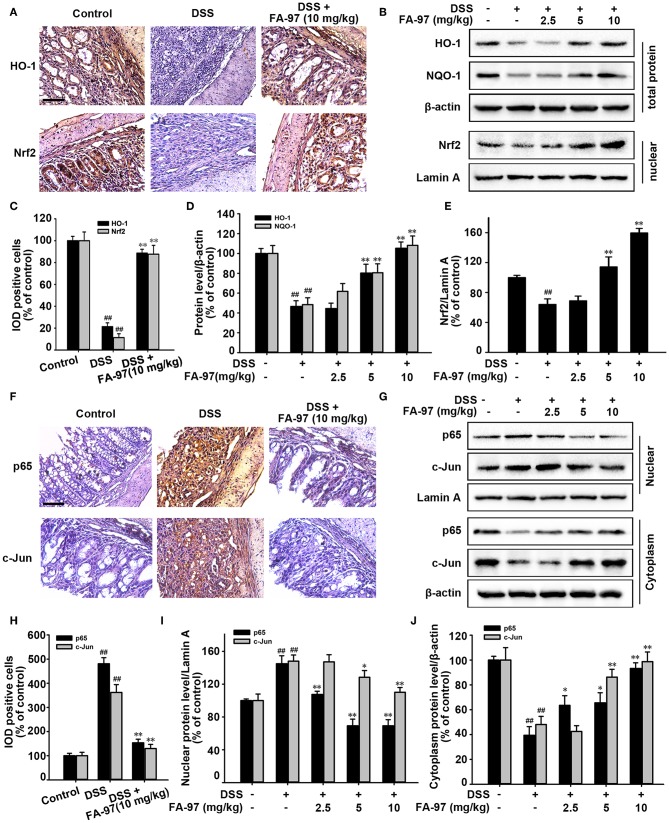
Effect of FA-97 on Nrf2/HO-1 signaling *in vivo*. **(A)** Representative images of immunohistochemical staining for HO-1 and Nrf2. **(B)** The expressions of HO-1, NQO-1 and Nrf2 in nuclear were determined by Western Blot. **(C)** Image pro plus software was used to quantify the IHC images and 10 fields were counted for each mouse. The integrated option density (IOD) of HO-1 and Nrf2 positive cells were shown. **(D,E)** Densitometric analysis was performed to determine the relative ratios of each protein. β-actin and Lamin A were used as nuclear and cytoplasmic markers, respectively. **(F)** The expressions of p65 and c-Jun in colonic tissues were detected by IHC and the positive cells were brown. **(G)** The expression of p65 and c-Jun were determined by Western Blot analysis. **(H)** Quantification of IHC images by image pro plus software and 10 fields were counted for each mouse. IOD of p65 and c-Jun positive cells were shown. **(I)** Densitometric analysis was performed to determine the relative ratios of nuclear proteins. Lamin A was used as nuclear marker. **(J)** Densitometric analysis was performed to determine the relative ratios of p65 and c-Jun in cytoplasm. β-actin was used as cytoplasmic marker. Each experiment was performed at least three times. Scale bars, 200 μm. Data were presented as means ± SD. ^##^*P* < 0.01 compared with control group and *P < 0.05, **P < 0.01 compared with DSS-treated group.

It has been reported that Nrf2/HO-1 signaling plays a vital role in protecting intestinal integrity by inducting the expression of variety detoxifying and antioxidant defense enzymes to reduce the intracellular ROS level, such as heme oxygenase-1 (HO-1), quinone oxidoreductase 1 (NQO1) and glutamate-cysteine ligase (GCL) (10). We therefore determined whether FA-97 exerted the antioxidative effect via intervening Nrf2 pathway. As expected, immunohistochemistry analysis showed that FA-97 (10 mg/kg) up-regulated the level of HO-1 and the Nrf2 nuclear translocation in colon tissues of DSS-induced colitis mice (Figures 5A,C). Moreover, Western Blot analysis showed that the total expression of HO-1 and NQO-1 in colon tissues of DSS-induced colitis mice were promoted by FA-97 (Figures 5B,D), as well as the nuclear Nrf2 level was also increased by FA-97 treatment (Figures 5B,E). Nrf2 is also reported as an anti-inflammatory factor relying on modulation of redox metabolism or crosstalk with several main inflammatory-related signaling, such as NF-κB and AP-1 pathways, as well as directly blocking the expression of IL-1β and IL-6 (30). We further to explore the effect of FA-97 on NF-κB and AP-1 signaling pathway in colitis mice. As shown in Figure 5F, compared to control group, there are more positive nuclear p65 and c-Jun staining detected in colon tissues of DSS-induced mice, while FA-97 (10 mg/kg) reduced the number of nuclear p65 and c-Jun positive cells (Figures 5F,H). In addition, Western Blot analysis showed that the expression of p65 and c-Jun in nuclear were both inhibited by FA-97 treatment (Figures 5G,I). Meanwhile, FA-97 increased the expression of cytoplasmic p65 and c-Jun (Figures 5G,J). In summary, FA-97 activated Nrf2/HO-1 signaling and inhibited the activation of NF-κB and AP-1 in DSS-induced colitis mice.

### FA-97 Relieves DSS-Induced Experimental Colitis Dependent on Nrf2/HO-1 Signaling

**Figure 6:**
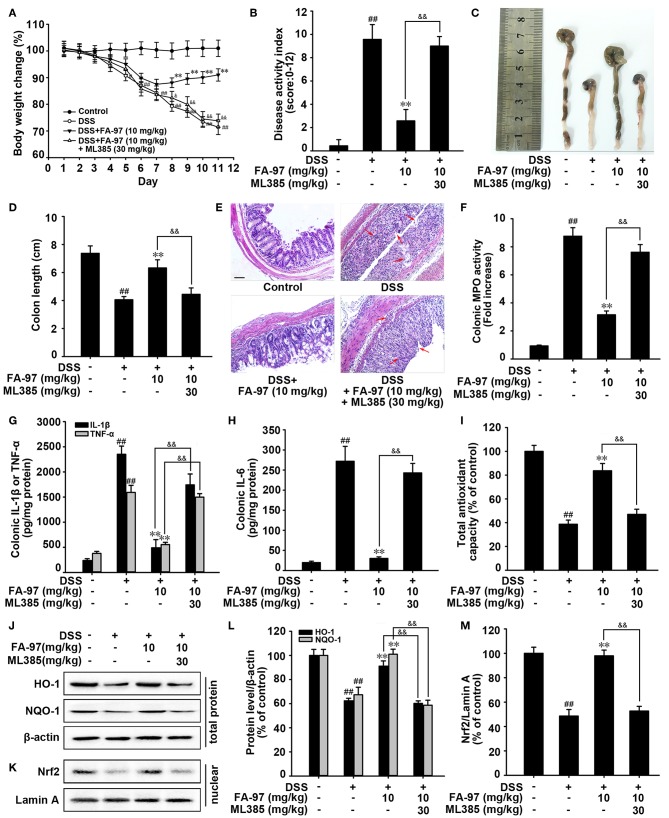
Nrf2/HO-1 signaling is critical for protection against DSS-induced colitis by FA-97. **(A)** Body weights of mice in each group (*n* = 8) were measured. **(B)** Disease activity index (DAI) of mice in each group was analyzed. **(C)** Macroscopic appearance of the representative colon from each group. **(D)** The quantification of colon length from each group of mice. **(E)** Representative images showing colon pathologic abnormalities with hematoxylin and eosin (H&E) staining. Scale bars, 100 μm. **(F)** The MPO activity in colonic tissues was detected by MPO Activity Assessment Kit using the O-dianisidine method. **(G,H)** The level of IL-1β, IL-6, and TNF-α in colonic homogenate were measured by ELISA. **(I)** The total antioxidant capacity in colon were measured according to the kit manufacturer's instructions. **(J,K)** The expression of HO-1, NQO-1, and Nrf2 in nuclear were determined by Western Blot. **(L,M)** Densitometric analysis was performed to determine the relative ratios of each protein. β-actin and Lamin A were used as nuclear and cytoplasmic markers, respectively. The results are representative of three independent experiments and expressed as means ± SD. ^##^*P* < 0.01 compared with control group, **P < 0.01 compared with DSS-treated group and ^&^*P* < 0.05, ^&&^*P* < 0.01 compared with DSS + FA-97-treated group.

To further confirm the critical role of Nrf2/HO-1 signaling in the protection effects of FA-97 on DSS-induced colitis, mice were administered ML385 (an Nrf2 specific inhibitor) concurrently with FA-97 treatment (30, 31). As shown in Figure 6A, monitoring changes in the body weight, FA-97 (10 mg/kg) could not decrease DSS-induced weight loss of mice treated with ML385 (30 mg/kg). The reduced DAI (Figure 6B) and improved colon shortening (Figures 6C,D) by FA-97 were withdrawn by ML383. In addition, ML385 reversed the protection effect of FA-97 on DSS-induced colon injury and the colon tissues of mice treated by ML385 concurrently with FA-97 were exhibited significant erosion of the intestinal mucosa and a great many inflammatory cells infiltration (Figure 6E). Moreover, the inhibition effects of FA-97 on DSS-induced colonic MPO activity (Figure 6F) and the main colonic pro-inflammatory factors (IL-1β, IL-6, and TNF-α) (Figures 6G,H) were attenuated by ML385, as well as FA-97-induced total antioxidant capacity was inhibited by ML385 (Figure 6I). Furthermore, the increased expression of HO-1 and NQO-1 in colons (Figures 6J,L) and FA-97-promoted nuclear Nrf2 level (Figures 6K,M) were abolished by ML385. Together, protection against DSS-induced colitis by FA-97 is dependent on Nrf2/HO-1 signaling.

### FA-97 Reduces LPS-Induced Pro-inflammatory Cytokine Production and Oxidative Stress *in vitro*

**Figure 7:**
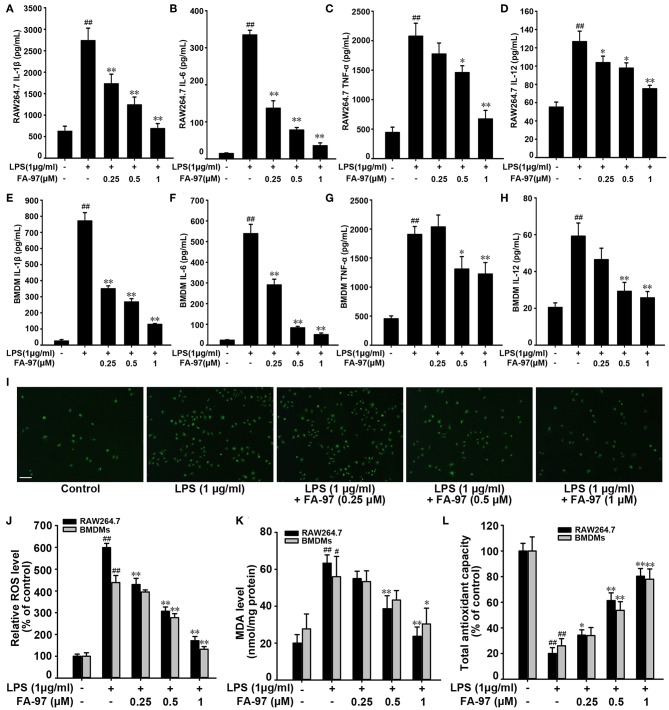
Effect of FA-97 on pro-inflammatory cytokine production and oxidative stress *in vitro*. RAW 264.7 cells and BMDMs were pretreated with FA-97 (0, 0.25, 0.5, and 1 μM) for 24 h followed by LPS (1 μg/ml) stimulation for another 2 h. The concentrations of IL-1β, IL-6, TNF-α and IL-12 in RAW 264.7 **(A–D)** and BMDMs **(E–H)** cell culture supernatants were measured by ELISA. **(I)** Representative images showing the DCFH-DA fluorescence in RAW264.7. **(J)** After treated with FA-97, RAW264.7 cells were collected and incubated with DCFH-DA for 30 min at 37°C in the dark, and then the level of ROS was measured by spectrofluorimeter. **(K)** The MDA level and the total antioxidant capacity **(L)** in RAW 264.7 cells and BMDMs were measured according to the kit manufacturer's instructions. Scale bars, 100 μm. The results are representative of three independent experiments and expressed as means ± SD. ^##^*P* < 0.01 compared with control group and *P < 0.05, **P < 0.01 compared with LPS-stimulated group.

To confirm our conclusion *in vivo*, we further investigated the effect of FA-97 on pro-inflammatory cytokine production and antioxidant defenses *in vitro* by using RAW 264.7 cells and bone marrow derived macrophages (BMDMs). The preliminary experiment showed that treatment with FA-97 ranging from 0.125 to 1 μM for 24 h had no effect on the cell viability of RAW 264.7 cells and BMDMs (Figure S2) and the reduced viability of BMDMs was observed at 2 μM FA-97. So the final concentration of FA-97 used in the following experiments was no more than 1 μM. ELISA assay showed that FA-97 inhibited LPS-induced secretion of IL-1β, IL-6, TNF-α, and IL-12 both in RAW 264.7 cells (Figures 7A–D) and BMDMs (Figures 7E–H). RT-PCR showed that the mRNA level of IL-1β, IL-6, TNF-α, and IL-12 in LPS-stimulated RAW 267.4 (Figure S3) and BMDMs (Figure S4) were reduced by FA-97 treatment. In addition, the LPS-induced DCFH-DA fluorescence in RAW 264.7 were inhibited by FA-97 (Figure 7I), supported by the ROS level detected by spectrofluorimeter (Figure 7J). We also found that the MDA level was increased by LPS both in RAW 264.7 cells and BMDMs, while FA-97 reduced the MDA level markedly (Figure 7K). Moreover, the total antioxidant capacity dramatically reduced by LPS was restored by FA-97 in a concentration-dependent manner (Figure 7L). Collectively, FA-97 inhibited the expression of pro-inflammatory cytokines and enhanced the antioxidant defenses both in RAW 264.7 cells and BMDMs.

### FA-97 Activates Nrf2/HO-1 Signaling *in vitro*

**Figure 8:**
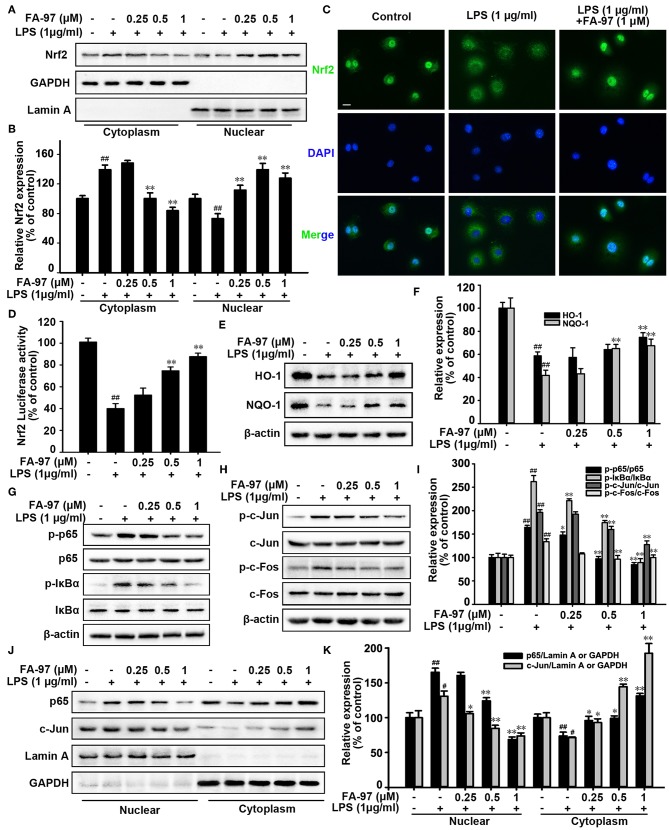
Effect of FA-97 on Nrf2/HO-1 signaling pathway *in vitro*. RAW264.7 cells were pretreated with FA-97 (0, 0.25, 0.5, and 1 μM) for 24 h followed by LPS (1 μg/ml) stimulation for 2 h. **(A)** The expression of Nrf2 in cytosolic and nuclear extracts were determined by Western Blot. Lamin A and GAPDH were used as nuclear and cytoplasmic markers, respectively. **(B)** Densitometric analysis was performed to determine the relative ratios of Nrf2. GAPDH and Lamin A were used as nuclear and cytoplasmic markers, respectively. **(C)** RAW 264.7 cell slides were immune-stained with anti-Nrf2 (green) and DAPI (blue), and then the nuclear translocation of Nrf2 was observed by confocal laser-scanning microscope. **(D)** After transfected with ARE luciferase reporter plasmid, the Nrf2 transcription activity of RAW 264.7 cells was detected by luciferase activity assay. **(E)** The level of HO-1, NQO-1, and β-actin were detected by Western Blot. **(F)** Densitometric analysis was performed to determine the relative ratios of HO-1 and NQO-1. **(G)** Protein level of p-p65, p65, p-IκBα, IκBα, and β-actin in RAW 264.7 cells were detected by Western Blot. **(H)** The protein level of p-c-Jun, c-Jun, p-c-Fos, c-Fos, and β-actin in RAW 264.7 cells were detected by Western Blot. **(I)** Densitometric analysis was performed to determine the relative ratios of each protein. **(J)** The expression of p65 and c-Jun in cytosolic and nuclear extracts of RAW 264.7 cells were determined by Western Blot. Lamin A and GAPDH were used as nuclear and cytoplasmic markers, respectively. **(K)** Densitometric analysis was performed to determine the relative ratios of p65 and c-Jun. GAPDH and Lamin A were used as nuclear and cytoplasmic markers, respectively. Scale bars, 20 μm. The results are representative of three independent experiments. ^#^*P* < 0.05, ^##^*P* < 0.01 compared with control group and *P < 0.05, **P < 0.01 compared with LPS-stimulated group.

To further elucidate the anti-oxidative mechanism of FA-97 *in vivo*, we investigated the effect of FA-97 on Nrf2/HO-1 pathway. Western Blot for nuclear separation showed that the nuclear Nrf2 level was increased by FA-97 (Figures 8A,B), which is supported by the immunofluorescence staining (Figure 8C) showing FA-97 (1 μM) increased the nuclear translocation of Nrf2. In addition, the transcription activity of Nrf2 was promoted by FA-97 in LPS-stimulated RAW264.7 cells conformed by the luciferase reporter assay (Figure 8D). Moreover, the total expression of both HO-1 and NQO-1 were increased by FA-97 in a concentration-dependent manner (Figures 8E,F). Based on the results of mechanism study *in vivo* (Figure 5), we then examined the effect of FA-97 on NF-κB and AP-1 signaling *in vivo*. As expected, LPS treatment increased the phosphorylation of p65, IκBα, c-Jun, and c-Fos, while FA-97 suppressed the expression of p-p65, p-IκBα, p-c-Jun, and p-c-Fos in LPS-induced RAW 264.7 cells (Figures 8G–I). Also, the Western Blot for nuclear separation showed that the nuclear expression of both p65 and c-Jun were inhibited by FA-97 (Figures 8J,K). Taken together, FA-97 activated Nrf2/HO-1 signaling and inhibited the phosphorylation and nuclear translocation of p65 and c-Jun *in vitro*.

### FA-97 Suppresses Oxidative Stress and Inflammation via Activating Nrf2/HO-1 Signaling *in vitro*

**Figure 9:**
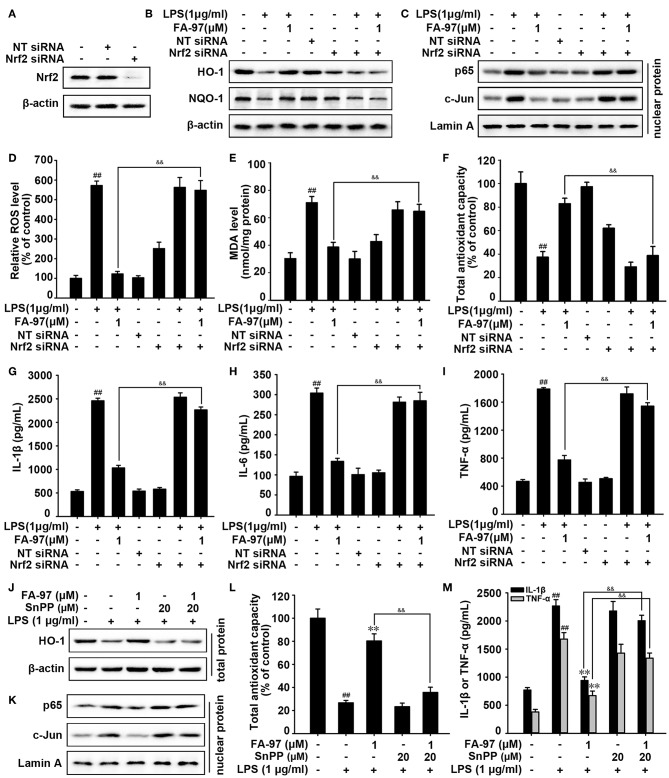
The Nrf2/HO-1 pathway was involved in the anti-oxidative and anti-inflammation effects of FA-97 in LPS-induced RAW 264.7 cells. After Nrf2 siRNA transfection, RAW 264.7 cells were treated with FA-97 (0, 0.25, 0.5, and 1 μM) for 24 h followed by LPS (1 μg/ml) stimulation for 2 h. **(A)** The expression of Nrf2 was detected by Western Blot after Nrf2 siRNA transfection. **(B)** The level of HO-1, NQO-1, and β-actin were detected by Western Blot. **(C)** The expression of p65 and c-Jun in nuclear extract were determined and Lamin A was used as nuclear marker. **(D)** The level of ROS was measured by spectrofluorimeter. The MDA level **(E)** and the total antioxidant capacity **(F)** in RAW 264.7 cells were measured according to the kit manufacturer's instructions. **(G–I)** The concentrations of IL-1β, IL-6, and TNF-α in RAW 264.7 cell culture supernatants were measured by ELISA. **(J–M)** RAW 264.7 cells were treated with FA-97 (1 μM) for 24 h followed by LPS (1 μg/ml) with or without SnPP (20 μM) stimulation for 2 h. The expression of HO-1 in cells was detected by Western Blot **(J)**, the expression of p65 and c-Jun in nuclear extract were determined and Lamin A was used as nuclear marker **(K)**. The total antioxidant capacity in cells were measured according to the kit manufacturer's instructions **(L)**, as well as the concentration of IL-1β and TNF-α in RAW 264.7 cell culture supernatants were measured by ELISA **(M)**. The results are representative of three independent experiments and expressed as means ± SD. ^##^*P* < 0.01 compared with control group, **P < 0.01 compared with LPS-stimulated group, ^&&^*P* < 0.01 compared with LPS + FA-97-treated group.

To explore whether the anti-oxidative effect of FA-97 is related to Nrf2, we diminished the expression of Nrf2 by transfecting Nrf2 siRNA. As shown in Figure 9A and Figure S5, compared with control group, the siRNA transfection lead to the lower expression of Nrf2 in RAW264.7 cells. HO-1 and NQO-1 proteins expression increased by FA-97 were withdrawn by Nrf2 siRNA transfection (Figure 9B and Figure S6A). Similarly, Nrf2 siRNA transfection reversed the down-regulated p65 and c-Jun in the nucleus by FA-97 treatment (Figure 9C and Figure S6B). In addition, the inhibitory effect of FA-97 on LPS-induced ROS generation and MDA level were attenuated by Nrf2 siRNA transfection (Figures 9D,E), as well as FA-97-induced total antioxidant capacity of LPS-treated RAW264.7 cells was inhibited by Nrf2 siRNA significantly (Figure 9F). Moreover, LPS-induced secretion of IL-1β, IL-6, and TNF-α in RAW 264.7 cells was decreased by FA-97 (1 μM), while the reduction was reversed by adding Nrf2 siRNA (Figures 9G–I).

Furthermore, a competitive inhibitor of HO-1 (SnPP) was used to investigate whether the anti-oxidative and anti-inflammation effects of FA-97 on LPS-induced RAW 264.7 cells were mediated through HO-1. The blockade of HO-1 by SnPP attenuated FA-97-induced expression of HO-1 (Figure 9J and Figure S7A), and FA-97-mediated inhibition of p65 and c-Jun expression in nucleus were reversed by SnPP (Figure 9K and Figure S7B). Besides, SnPP significantly inhibited FA-97-induced total antioxidant capacity (Figure 9L), and the inhibitory effects of FA-97 on ROS generation (Figure S8) or MDA level (Figure S9) in LPS-induced RAW 264.7 cells were attenuated by SnPP. In addition, the decreased secretion of IL-1β and TNF-α (Figure 9M), as well as IL-6 (Figure S10) in RAW 264.7 cells by FA-97 were abolished by SnPP. Together, FA-97 exerted the anti-oxidative and anti-inflammation effects by activating Nrf2/HO-1 signaling.

## Discussion

Recently, targeting the excessive activity of the adaptive immune system by using biological agents such as monoclonal antibodies against TNF-α, is the most popular approach in IBD treatment (32). However, although these anti-TNF-α agents are successful in treating many patients, only a third or less will achieve remission and many of those who do will eventually lose their response (33). Therefore, it is urgent to explore novel preventive intervention and therapeutics with high efficacy and few side effects for IBD.

In this study, FA-97 (caffeic acid phenethyl ester 4-*O*-glucoside), a new synthetic caffeic acid phenethyl ester (CAPE) derivative is synthesized (Figure 1) and proved to ameliorate DSS-induced colitis against oxidative stress by activating Nrf2/HO-1 pathway *in vivo* and *in vitro*. As a phenolic constituent derived from honeybee propolis, CAPE possesses great anti-inflammatory activities and can suppress the pro-inflammatory cytokines production and enhance epithelial barrier function, which could be a potential therapeutic agent for IBD (17, 18). However, the unstable chemical property, low water solubility and the poor bioavailability of CAPE limit its efficacy (19–21). Therefore, in order to enhance the water solubility, chemical stability and pharmacological activity of CAPE, FA-97 was newly synthesized by introducing a D-glucose into CAPE to construct a glucosidic bond (Figure 1). In the synthesizing process, the D-glucose was protected with acetyl group and then brominated with HBr/CH_3_COOH. The brominated D-glucose was then coupled with CAPE under the catalysts EDCI and HOBT. With the deprotection of coupled key intermediate, FA-97 was synthesized in good yields.

On account of intestinal inflammation induced by DSS is similar to the pathological characterization of human IBD, DSS-induced colitis mice are commonly adopted in the progress of drug discovery (24). Therefore, to evaluate the protective effect of FA-97 on IBD *in vivo*, we established DSS-induced colitis mice model here. As 5-ASA is effective in preventing humans colitis, we investigated the efficacy of FA-97 using CAPE and 5-ASA as references. We found that FA-97 attenuated the intestinal injury and symptomatic features of DSS-induced colitis mice, including body weight loss, colon length shortening, spleen swelling and serious colonic tissue damage (Figure 2). FA-97 also reversed the inflammatory cell infiltration in colon tissues, which were monitored by CD11b expression, MPO and iNOS activities. However, CAPE showed no effect on the CD11b expression and no apparent effect on MPO and iNOS activities (Figure 3). In addition, FA-97 successfully prevented colitis by inhibiting the production of IL-1β, IL-6, TNF-α, IL-12, MIP-1α, and IL-17 in colons. Furthermore, the systemic toxicity of FA-97 *in vivo* was evaluated. As a result, H&E staining of major organs of mice in control and FA-97-treated groups did not show remarkable abnormality (Figure S11). In addition, there were no notable changes in the analyzed hematological parameters from FA-97-treated mice (Figure S12), as well as the liver enzyme profiles in plasma (ALT, AST, and Urea nitrogen) were also not changed by FA-97 treatment (Figure S13). Thus, FA-97 has a safety profile *in vivo* and might be a promising candidate for colitis therapy.

Over the past few years, many studies have established a direct relation between oxidative stress and the development of IBD in human and experimental colitis mice. Clinical studies showed that the level of ROS, products of lipid peroxidation and modification of proteins were increased in the intestinal mucosa of UC or CD patients (34), as well as these highly cytotoxic molecules contribute to tissue damage in IBD (35); the reduced antioxidant level has been observed in the intestinal mucosa and peripheral red blood cells of IBD patients (36); genetic polymorphisms in antioxidant enzymes are closely related to the changed enzyme activity and a higher risk of IBD (37). Preclinical studies showed that there was an increased ROS formation in the colonic mucosa from colitis mice (38); experimental colitis is in accordance with a decrease of endogenous antioxidants in colonic tissue (39); ROS-detoxifying enzymes deletion by gene knockout increases the susceptibility to tissue destruction (40), while overexpression of antioxidant enzymes attenuated colitis in mice (41). Moreover, there are several anti-oxidative drugs have been successfully used in IBD (42). Therefore, imbalance the oxidation and anti-oxidation mechanisms in IBD may be a therapeutic strategy with great promise. In this study, we found that FA-97 reduced the ROS production and MDA level, as well as promoted the total antioxidant capacity both in DSS-induced colitis mice and LPS-stimulated RAW 264.7 cells. Our findings indicated that FA-97 exhibited the protection effect on colitis via counteracting oxidative stress and enhancing the antioxidant defenses.

Nrf2/HO-1 signaling orchestrates key cellular antioxidant response mechanisms, which has become an attractive target for preventing and treating several inflammatory diseases including IBD (10). Over the last few decades, the protective role of Nrf2 activation has been established in the treatment of IBD (14, 16), as well as numerous Nrf2 activators have been developed and what's more notable is that some of them are undergoing clinical trials currently (10, 43). Besides, CAPE has been reported as a known activator of HO-1 expression (17). To explore the mechanism of FA-97 ameliorates colitis, we then detected the effect of FA-97 on Nrf2/HO-1 pathway. The nucleus translocation of Nrf2 is the key step in activating the expression of cytoprotective phase II detoxification and antioxidant enzymes (9). Therefore, the Nrf2 level in nuclear and its nucleus translocation were detected initially. As a result, we found that nucleus level of Nrf2 was up-regulated by FA-97, following by the increased expression of HO-1 and NQO-1 both in colon tissues and LPS-induced RAW 264.7 cells. Moreover, the transcription activity of Nrf2 in RAW 264.7 cells was promoted in a concentration-dependent manner.

Along with a master regulator of redox homeostasis, Nrf2 also induces an anti-inflammatory phenotype and acts as an anti-inflammatory factor by blocking the transcription of the pro-inflammatory genes (IL-1β and IL-6) directly, as well as modulating redox metabolism or the main inflammatory-related signaling pathways indirectly including NF-κB and AP-1 (43–45). The activated NF-κB signaling pathway has been found both in DSS-induced colitis animals and IBD patients (46). Moreover, the nuclear transcription factor AP-1 can also trigger the expression of pro-inflammatory cytokines in IBD progression, which is consisting of hetero or dimeric complexes by c-Jun and c-Fos (45). Therefore, to further explore the mechanism of FA-97, the activation of p65, c-Jun, and c-Fos were then evaluated. Our data showed that FA-97 inhibited the nucleus expression of p65 and c-Jun in DSS-induced colitis mice. Meanwhile, the phosphorylation and nuclear translocation of p65, c-Jun, and c-Fos in LPS-induced RAW 264.7 cells were inhibited by FA-97. Taken together, FA-97 activates Nrf2/HO-1 followed by the inhibition of NF-κB/AP-1 signaling pathways, which could be a potential Nrf2 activator.

To further confirm the induction of Nrf2/HO-1 is responsible for the protection effects of FA-97 on DSS-induced colitis, ML385 (an Nrf2 inhibitor) was used *in vivo*. The attenuated the profound weight loss, reduced DAI, improved colon shortening and colon injury remission of mice treated with FA-97 were withdrawn by ML383. Besides, the inhibited MDA level and the decreased pro-inflammatory factors with FA-97 treatment were abolished by ML385, as well as ML385 inhibited FA-97-induced total antioxidant capacity and FA-97-increased expression of HO-1, NQO-1 and Nrf2. These findings support that protection against DSS-induced colitis by FA-97 is dependent on Nrf2/HO-1 signaling *in vivo*. Furthermore, to further verify the role of Nrf2/HO-1 in the anti-inflammation and anti-oxidation mechanism of FA-97 *in vitro*, we transfected RAW 264.7 cells with Nrf2 siRNA and a competitive inhibitor of HO-1 (SnPP) was used. As a result, Nrf2 siRNA transfection or SnPP reversed the down-regulated p65 and c-Jun in nucleus by FA-97 treatment, which means the inhibition effects of FA-97 on NF-κB and AP-1 signaling is dependent of Nrf2 activation and HO-1 expression. Moreover, the modulation effect of FA-79 on LPS-induced ROS generation, MDA level and the total anti-oxidant capacity, as well as the pro-inflammatory cytokines secretion were reversed both by the Nrf2 siRNA and Snpp, indicating FA-97 exerts the antioxidant an anti-inflammation effects by activating Nrf2/HO-1 signaling pathway. In summary, Nrf2//HO-1 is involved in the antioxidant and anti-inflammation effects of FA-97 *in vivo* and *in vitro*.

**Figure 10:**
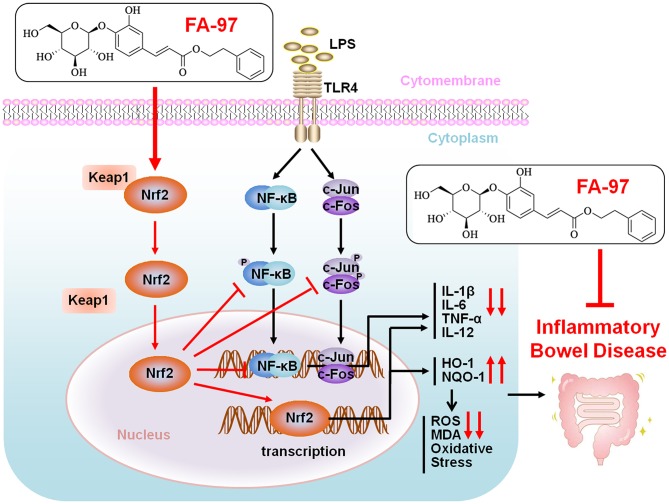
Proposed mechanistic model of FA-97 ameliorates DSS-induced colitis against oxidative stress by activating Nrf2/HO-1 pathway. Our findings demonstrated a scenario where FA-97 activates Nrf2 and promotes its nuclear translocation, on one hand increasing the expression of its downstream target proteins HO-1 and NQO-1, to reduce the ROS level and enhance the oxidant resistance, on the other hand inhibiting the NF-κB and AP-1 signaling to suppress the expression of pro-inflammatory cytokines (IL-1β, IL-6, TNF-α, and IL-12), and eventually meliorates inflammatory bowel disease.

In the present study, we elucidates FA-97, a new synthetic CAPE derivative, meliorates DSS-induced colitis against oxidative stress and LPS-induced pro-inflammatory cytokine expression in RAW264.7 cells and BMDMs by activating Nrf2/HO-1 signaling. Our findings demonstrated a scenario where FA-97 activates Nrf2 and promotes its nuclear translocation, on one hand increasing the expression of its downstream target proteins HO-1 and NQO-1, to reduce the ROS level and enhance the oxidant resistance, on the other hand inhibiting the NF-κB and AP-1 signaling to suppress the expression of pro-inflammatory cytokines (IL-1β, IL-6, TNF-α, and IL-12), and eventually meliorates inflammatory bowel disease (Figure 10). However, there are some limitations in the present study. Along with the antioxidant or anti-inflammation effect, effects of FA-97 on the intestinal flora and immune system should be evaluated by more IBD mice models in our further study. According to our preliminary study, FA-97 can also inhibit DSS-induced leakiness of the colon and maintain the epithelial barrier function. Whether the increased antioxidant ability or lowered cytokine expression in the whole colon tissue after FA-97 treatment is the cause or consequence of the indeed improved epithelial barrier function remains to be further elucidated, as well as whether FA-97 could intervene other signaling pathway and the exact activation mechanism of FA-97 on Nrf2/HO-1 pathway needs deeper exploration.

In conclusion, we demonstrated that FA-97, a new synthetic CAPE derivative, meliorates DSS-induced colitis against oxidative stress *in vivo* and LPS-stimulated pro-inflammatory cytokine expression in RAW264.7 cells and BMDMs *in vitro*. This effect is associated with the inhibition of oxidative stress via the activation of Nrf2/HO-1 signaling. FA-97 could be a potential agent as a candidate for IBD therapy.

## Data Availability Statement

The data used to support the findings of this study are available from the corresponding author upon request.

## Ethics Statement

The animal study was reviewed and approved by Animal Ethics Committee of Guangzhou University of Chinese Medicine.

## Author Contributions

YH and QW designed this study. YH wrote the manuscript. ZW synthesized the compounds. YM, YZ, and TW performed *in vivo* experiments. YX and WH performed *in vitro* experiments. YL and JX analyzed the data. XB assisted with the manuscript modification. All authors read and approved the final manuscript.

### Conflict of Interest

The authors declare that the research was conducted in the absence of any commercial or financial relationships that could be construed as a potential conflict of interest.
