# Supplementary material for: FA-97, a New Synthetic Caffeic Acid Phenethyl Ester Derivative, Ameliorates DSS-Induced Colitis Against Oxidative Stress by Activating Nrf2/HO-1 Pathway
Source: Front Immunol. 2020 Jan 8;10:2969. doi: 10.3389/fimmu.2019.02969 (PMC6960141; doi:10.3389/fimmu.2019.02969)
Supplement: Supplementary file 1 [file Data_Sheet_1.pdf]

Supplementary Data

Title: FA-97, a new synthetic caffeic acid phenethyl ester derivative, ameliorates DSS-induced colitis against oxidative stress by activating Nrf2/HO-1 pathway

***To whom correspondence should be addressed:**

Yujie Huang, PhD

Institute of Clinical Pharmacology

Guangzhou University of Chinese Medicine.

No 12, Jichang Road, Guangzhou, Guangdong 510006, China.

Email: huangyujie@gzucm.edu.cn

Phone: 86-20-36585404

Supplementary data contains six supplementary figures (Fig. S1-S13).

Figure S1.

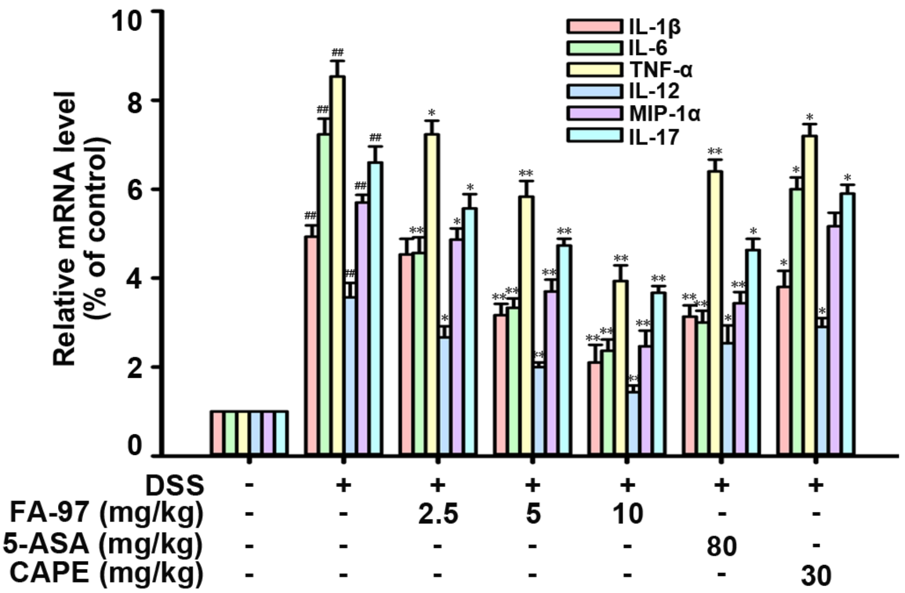

Supplementary Figure S1. The mRNA level of IL-1β, IL-6, TNF-α, IL-12, MIP-1α and IL-17 in colonic tissues was detected by RT-PCR. Each experiment was performed at least three times. Data are presented as mean ± SD. $^{\#\#}P < 0.01$ compared with control group and $^{*}P < 0.05$, $^{**}P < 0.01$ compared with DSS-treated group.

Figure S2.

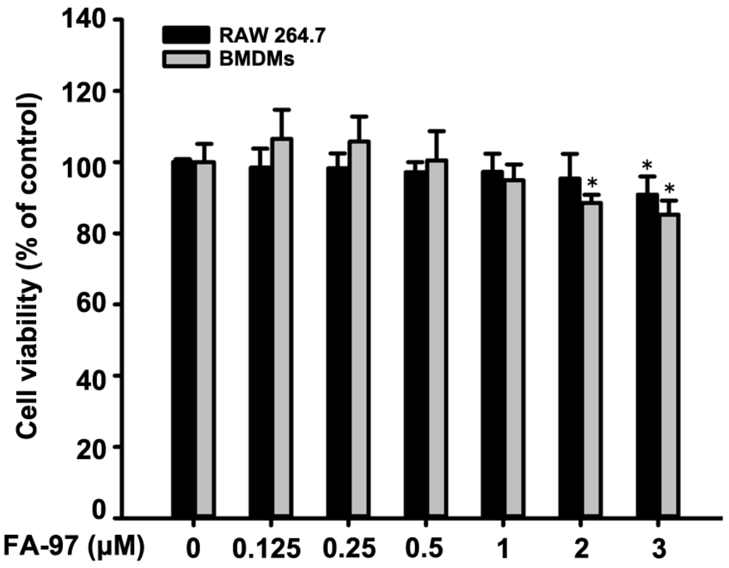

Supplementary Figure S2. Effect of FA-97 on cell viability of RAW 264.7 cells and bone marrow derived macrophages (BMDMs). Cells were plated into 96-well plates (2×10^5 cells/well) with fresh medium overnight, then treated with FA-97 (0, 0.125, 0.25, 0.5, 1, 2, 3 μM) for 24 h. Cell viability was detected by CCK8 assay. Experiment was performed at least three times and data are presented as mean ± SD. $^{*}P < 0.05$ compared with control group.

Figure S3.

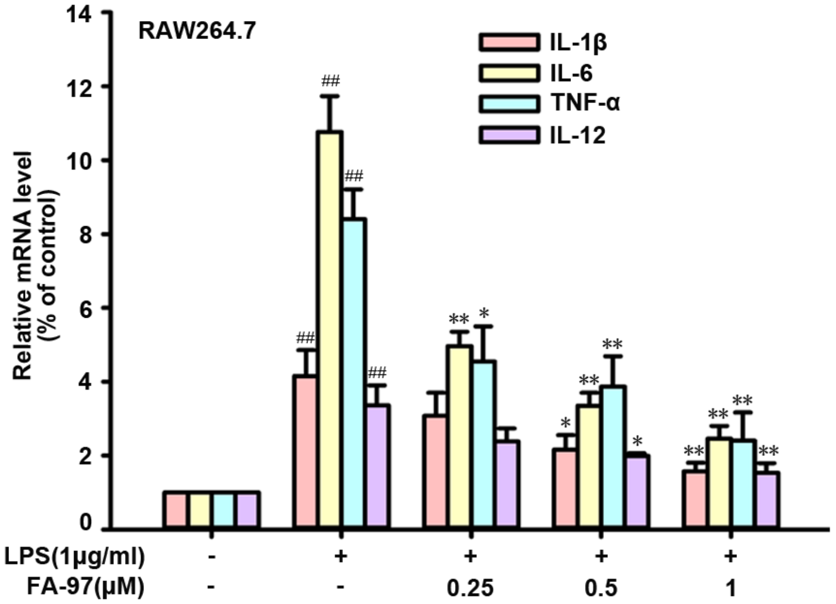

Supplementary Figure S3. RAW 264.7 cells were pretreated with FA-97 (0, 0.25, 0.5 and 1 μM) for 24 h followed by LPS (1 μg/ml) stimulation for another 2 h. The mRNA levels of IL-1β, IL-6, TNF-α and IL-12 in RAW 264.7 cells were measured by RT-PCR. Data are presented as mean ± SD. $^{\#\#}P < 0.01$ compared with control group and $^{*}P < 0.05$, $^{**}P < 0.01$ compared with LPS-stimulated group.

Figure S4.

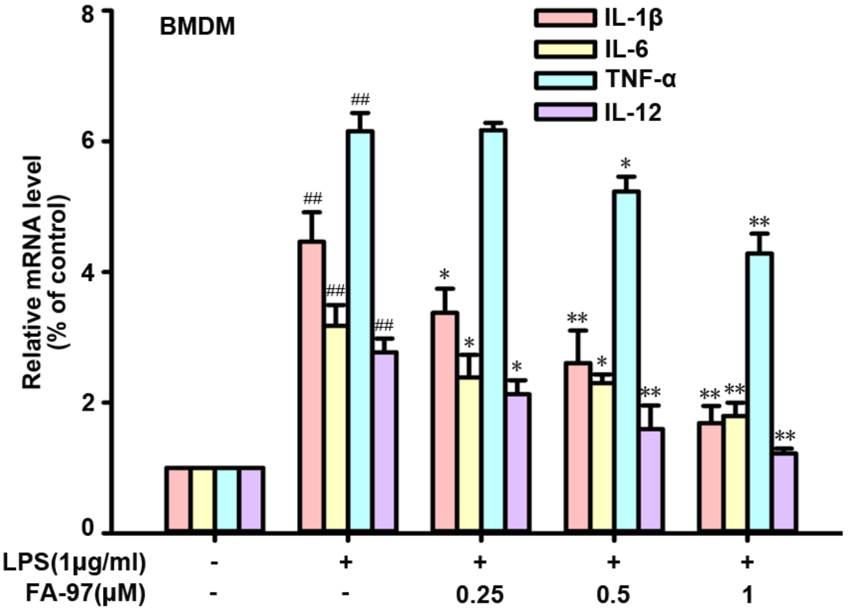

Supplementary Figure S4. After isolated from the bone marrow, the primary bone marrow derived macrophages (BMDMs) were pretreated with FA-97 (0, 0.25, 0.5 and 1 μM) for 24 h followed by LPS (1 μg/ml) stimulation for another 2 h. The mRNA levels of IL-1β, IL-6, TNF-α and IL-12 in BMDMs were measured by RT-PCR. Data are presented as mean ± SD. $^{\#\#}P < 0.01$ compared with control group and $^{*}P < 0.05$, $^{**}P < 0.01$ compared with LPS-stimulated group.

Figure S5.

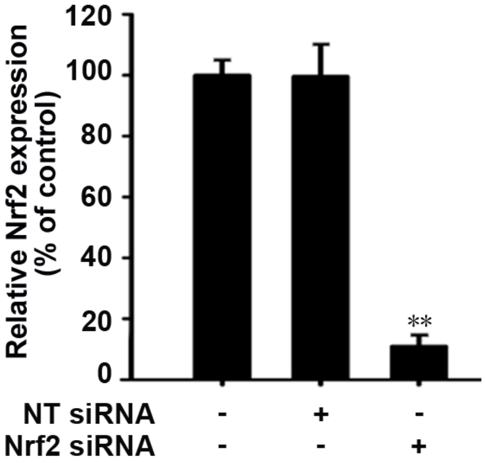

Supplementary Figure S5. Densitometric analysis was performed to determine the relative ratios of Nrf2 (related to Figure 9A). The expression of Nrf2 in RAW 264.7 cells was detected by Western Blot after Nrf2 siRNA or non-targeting siRNA (NT siRNA) transfection. The results are representative of three independent experiments and expressed as means ± SD. $^{**}P < 0.01$ compared with NT siRNA transfection group.

Figure S6.

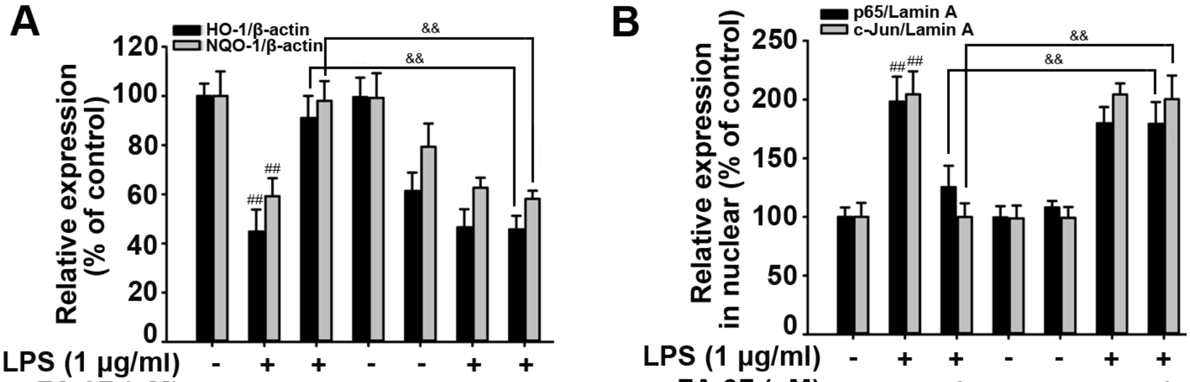

Supplementary Figure S6. The relative ratios of HO-1, NQO-1/β-actin (A), and p65, c-Jun/LaminA (B) were presented by densitometric analysis (related to Figure 9B-C). The expression of HO-1, NQO-1, β-actin, p65, c-Jun and Lamin A in RAW 264.7 cells was detected by Western Blot after Nrf2 siRNA or non-targeting siRNA (NT siRNA) transfection. Lamin A was used as nuclear marker. The results are representative of three independent experiments and expressed as means ± SD. $^{\#\#}P <$ 0.01 compared with control group, $^{**}P < 0.01$ compared with LPS-stimulated group, $^{\&\&}P < 0.01$ compared with LPS + FA-97-treated group.

Figure S7.

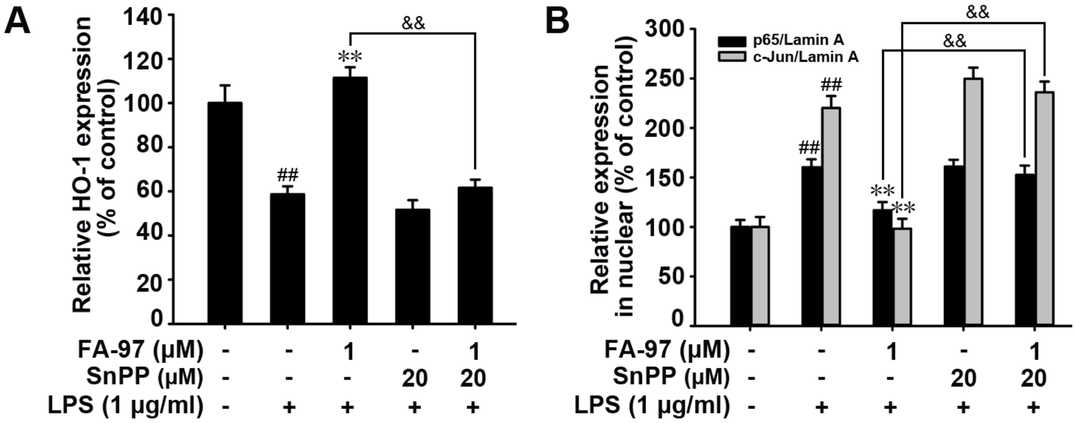

Supplementary Figure S7. The relative ratios of HO-1/β-actin (A), and p65, c-Jun/LaminA (B) were presented by densitometric analysis (related to Figure 9J-K). RAW 264.7 cells were treated with FA-97 (1 μM) for 24 h followed by LPS (1 μg/ml) with or without SnPP (20 μM) stimulation for 2 h, and then the expression of HO-1, β-actin, p65, c-Jun and Lamin A was detected by Western Blot. Lamin A was used as nuclear marker. The results are representative of three independent experiments and expressed as means ± SD. $^{##}P < 0.01$ compared with control group, $^{**}P < 0.01$ compared with LPS-stimulated group, $^{\&\&}P < 0.01$ compared with LPS + FA-97-treated group.

Figure S8.

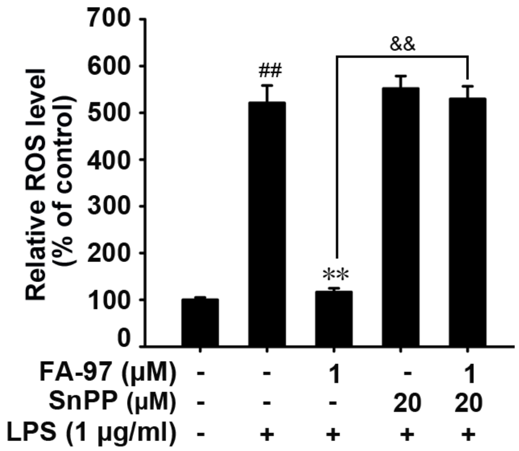

Supplementary Figure S8. Effect of SnPP on ROS level level in RAW 264.7 cells. RAW 264.7 cells were treated with FA-97 (1 μM) for 24 h followed by LPS (1 μg/ml) with or without SnPP (20 μM) stimulation for 2 h. After cells were incubated with DCFH-DA for 30 min at 37 ℃ in the dark, ROS level was measured by spectrofluorimeter. Results are representative of three independent experiments and expressed as means ± SD. $^{##}P < 0.01$ compared with control group, $^{**}P < 0.01$ compared with LPS-stimulated group, $^{\&\&}P < 0.01$ compared with LPS + FA-97-treated group.

Figure S9.

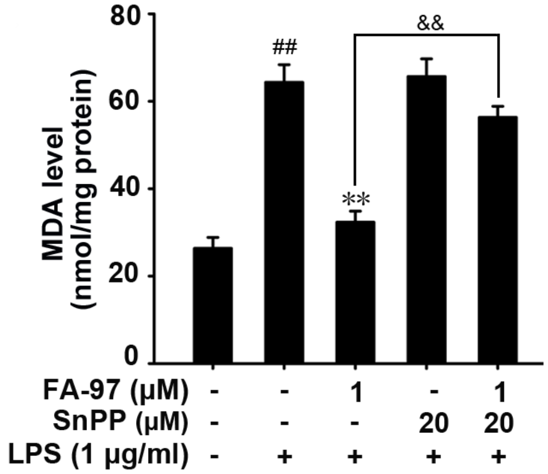

Supplementary Figure S9. Effect of SnPP on the MDA level in RAW 264.7 cells. RAW 264.7 cells were treated with FA-97 (1 μM) for 24 h followed by LPS (1 μg/ml) with or without SnPP (20 μM) stimulation for 2 h. The MDA level was measured according to the kit manufacturer's instructions. Results are representative of three independent experiments and expressed as means ± SD. $^{\#\#}P < 0.01$ compared with control group, $^{**}P < 0.01$ compared with LPS-stimulated group, $^{\&\&}P < 0.01$ compared with LPS + FA-97-treated group.

Figure S10.

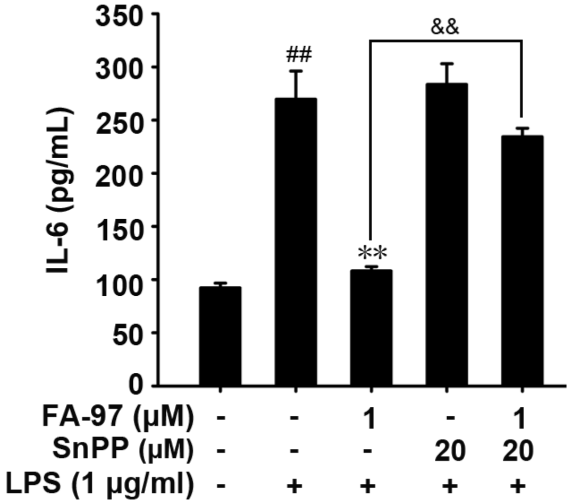

Supplementary Figure S10. Effect of SnPP on IL-6 secretion of RAW 264.7 cells. RAW 264.7 cells were treated with FA-97 (1 μM) for 24 h followed by LPS (1 μg/ml) with or without SnPP (20 μM) stimulation for 2 h. The concentration of IL-6 in RAW 264.7 cell culture supernatants were measured by ELISA assay. Results are representative of three independent experiments and expressed as means ± SD. $^{\#\#}P < 0.01$ compared with control group, $^{**}P < 0.01$ compared with LPS-stimulated group, $^{\&\&}P < 0.01$ compared with LPS + FA-97-treated group.

Figure S11.

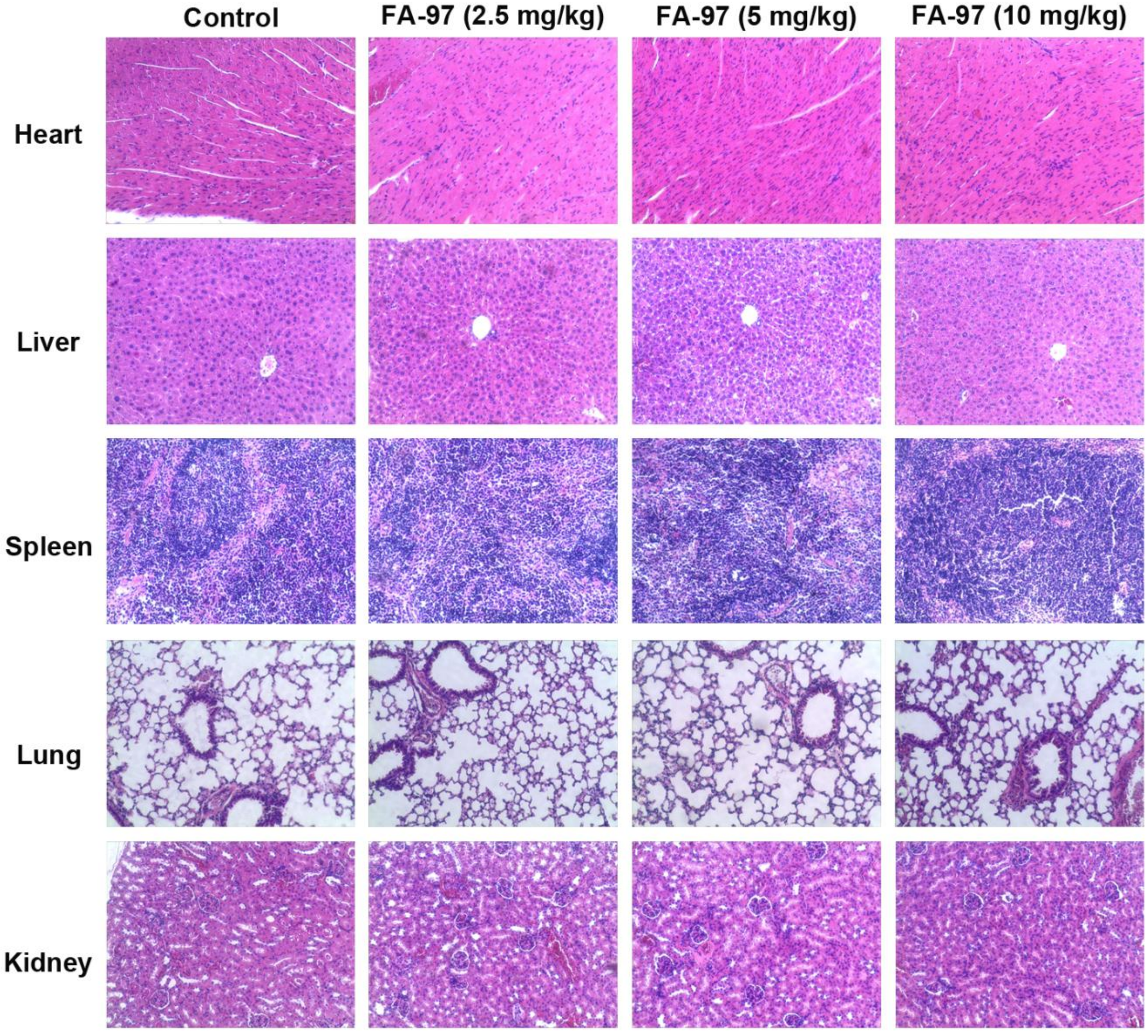

Supplementary Figure S11. Toxicity and safety profile of FA-97 in C57BL/6 mice. Heart, liver, spleen, lung and kidney from mice in each group were harvested, immersed in 4% formaldehyde (pH 7.4) for 24 h, embedded in paraffin, cut into sections and stained with hematoxylin & eosin (H&E). The pictures are representative from control and FA-97 (2.5, 5 and 10 mg/kg)-treated groups.

Figure S12.

Hematological parameters	Control	FA-97 (2.5 mg/kg)	FA-97 (5 mg/kg)	FA-97 (10 mg/kg)	Standard
White blood cells (10^9/L)	9.12±1.25	8.56±2.01	9.86±1.98	9.08±1.77	4.53--9.99
Red blood cells (10^{12}/L)	15.02±0.36	13.56±1.07	14.88±1.89	15.22±1.09	9.59--16.49
Hemoglobin (g/L)	149±5	155±8	160±10	155±5.2	128--161
Platelet	824±32	621±5.2	707±51	847±23	579--1066
Lymphocytes (10^9/L)	15.66±3.25	23.36±5.22	18.69±4.21	15.89±3.01	5--20
Monocytes (10^9/L)	2.5±0.6	2.13±0.65	2.25±0.58	2.11±0.16	2--6
Eosinophils (10^9/L)	0.006±0.006	0.006±0.006	0.006±0.006	0.006±0.006	0.4--8.0
Basophils (10^9/L)	0.025±0.005	0.035±0.005	0.015±0.005	0.020±0.005	0--1
Mean corpuscular volume (fL)	52.15±0.7	55.33±2.1	57.98±3.2	50.68±1.4	41--60
Hematocrit (%)	49.87±1.9	41.56±3.2	45.56±2.35	46.78±2.69	34--50
Mean corpuscular hemoglobin (pg)	16.55±0.08	15.21±0.55	16.23±0.05	14.88±0.09	13--19

Supplementary Figure S12. Effect of FA-97 on the hematological parameters of C57BL/6 mice. The hematological parameters of C57BL/6 mice in the control and FA-97 (2.5, 5 and 10 mg/kg)-treated groups were detected. The standards were determined by measuring blood hematological parameters of 40 healthy female C57BL/6 mice in the same living conditions with the experimental animals.

Figure S13.

Hematological biochemical parameters	Control	FA-97 (2.5 mg/kg)	FA-97 (5 mg/kg)	FA-97 (10 mg/kg)	Standard
Alanine aminotransferase (ALT,U/L)	35.6±2.2	29.6±1.3	31±5.4	35±6.7	21.8--44.7
Aspartate aminotransferase (AST,U/L)	110.5±5.6	98.3±12.5	95.8±6.6	82±10.2	45.6--120.8
Urea nitrogen (nmol/L)	8.5±1.56	11.02±0.45	9.56±0.68	10.2±0.5	9.2--13.5

Supplementary Figure S13. Effect of FA-97 on the biochemical parameters of C57BL/6 mice. The biochemical parameters of C57BL/6 mice in the control and FA-97 (2.5, 5 and 10 mg/kg)-treated groups were detected. The standards were determined by measuring blood biochemical parameters of 40 healthy female C57BL/6 mice in the same living conditions with the experimental animals.
